# A Web-Based Sexual Violence, Alcohol Misuse, and Bystander Intervention Program for College Women (RealConsent): Randomized Controlled Trial

**DOI:** 10.2196/43740

**Published:** 2023-06-21

**Authors:** Laura Francisca Salazar, Anne Marie Schipani-McLaughlin, Yesser Sebeh, Zainab Nizam, Matt Hayat

**Affiliations:** 1 Department of Health Policy and Behavioral Science School of Public Health Georgia State University Atlanta, GA United States; 2 Department Population Health Sciences School of Public Health Georgia State University Atlanta, GA United States

**Keywords:** gender-based violence, prospective study, randomized controlled trial, rape or prevention and control, rape or statistics and numerical data, school based, violence, alcohol, women, efficacy, program, intervention, behavior

## Abstract

**Background:**

Sexual violence (SV) incidence among college women has been invariant for the past 20 years. Innovative prevention strategies that are low resource and technology driven but demonstrate efficacy are greatly needed.

**Objective:**

The aim of this study was to determine the efficacy of a novel theoretically driven internet-based intervention for first-year college students who identify as women (RealConsent) in reducing their risk of exposure to SV and alcohol misuse as well as increasing alcohol protective and bystander behaviors.

**Methods:**

This randomized controlled trial involved first-year college students who identified as women (n=881) attending 1 of 3 universities in the southeastern United States. Participants aged 18 to 20 years were randomized to RealConsent (444/881, 50.4%) or to an attention-matched placebo control (437/881, 49.6%). RealConsent is fully automated and consists of four 45-minute modules that incorporate entertainment-education media and proven behavior change techniques. The primary outcome was exposure to SV; the secondary outcomes were alcohol protective behaviors, dating risk behaviors, alcohol misuse, and bystander behavior. Study outcomes were assessed at baseline and 6-month follow-up.

**Results:**

Among participants with some exposure to SV, those in the RealConsent group experienced less exposure to SV than the placebo group (adjusted incidence rate ratio 0.48, 95% CI 0.33-0.69; *P*=.002). Furthermore, participants in the RealConsent group engaged in more alcohol protective behaviors (adjusted odds ratio 1.17, 95% CI 0.12-2.22; *P*=.03) and were less likely to binge drink (adjusted incidence rate ratio 0.81, 95% CI 0.67-0.97; *P*=.003). Finally, participants in the RealConsent group who had 100% dosage were more likely to engage in bystander behavior than those with <100% dosage plus placebo group (adjusted odds ratio 1.72, 95% CI 1.17-2.55; *P*=.006).

**Conclusions:**

A comprehensive exposure to SV, alcohol use, and bystander educational program was successful in decreasing the occurrence of exposure to SV among those most at risk and in increasing alcohol protective behaviors. Because of its web-based and mobile technologies, RealConsent can be easily disseminated and holds potential for reducing campus SV.

**Trial Registration:**

ClinicalTrials.gov NCT03726437; https://clinicaltrials.gov/ct2/show/NCT03726437

## Introduction

### Background

Sexual violence (SV) is a widespread, complex social and behavioral problem for which we currently have few comprehensive approaches to prevention and even fewer for college women [1-4]. SV encompasses a range of behaviors on a continuum from *minor* behaviors (eg, catcalling and verbal suggestions of intent to force someone to have sex) to more extreme behaviors (eg, attempted or completed rape). An estimated 1 in 5 women report having experienced attempted or completed rape in their lifetime, and 43.9% have experienced other forms of SV in their lifetime [5,6]. Consequences of SV include posttraumatic stress disorder, suicidality, problematic alcohol use, and increased risk for re-exposure to SV [7,8]. College campuses, in general, are high-risk environments for SV. In 2019, about 26% of college women experienced SV, which represents a 3% increase from 2015 [9,10]. SV largely occurs during a woman’s freshman or sophomore year of college and most often (75%-80% of the time) at the hands of a known assailant [11]. Alcohol use is also highly prevalent on college campuses: >60% of US college students drink alcohol, and 35% engage in heavy episodic drinking (≥5 drinks for men and ≥4 drinks for women in 1 sitting) [12]. It is not surprising then that alcohol is a key contributor to SV; 50% to 79% of SV incidents involve alcohol use by the SV survivor, perpetrator, or both [11,13-16]. Despite decades of research, we have not moved the needle on campus SV [11,17,18].

Although numerous existing prevention programs focus on reducing perpetration rates among men or improving bystander behaviors, there is a need for programs that combine multiple, comprehensive prevention strategies [19-22]. In addition, in the current intervention landscape, there is a lack of programs targeted specifically at educating and empowering women. Although the majority of programs do and *should focus* on identifying and preventing risk for perpetration of SV by men, there are actionable tools and skill sets that can equip women to advocate for themselves when entering the college landscape [20,23-25]. Protective drinking behavior is a teachable skill set that has been identified as a protective factor for exposure to SV [26-31]. Programs directed at women should also seek to reduce risk by increasing individual ability to perceive risk for exposure to SV, identify dangerous dating situations, enhance self-defense skills, and improve assertive communication skills. Although some programs do currently exist that address alcohol as a risk factor for SV and protective drinking behaviors [23,27,32], there are no programs specifically designed for college women that include alcohol use as a central program component. Furthermore, most prevention programs for both men and women are delivered through in-person small group settings, which limits the scope of dissemination.

### RealConsent (Women's Version)

RealConsent (women's version) was developed using web-based and mobile technologies and is an educational program aimed specifically at helping college-age women develop protective behaviors against SV. Building off the delivery model of the 2014 RealConsent program aimed at college-age men [33], the women's version is meant to promote women’s abilities to understand and perceive risk for exposure to SV; help women to understand the dangers of, and protective behaviors for, alcohol consumption; and teach women how to leverage peer networks as bystanders able to intervene in dangerous situations. In this study, RealConsent was evaluated for efficacy in reducing SV incidence, affecting alcohol and dating risk and protective factors, reducing alcohol misuse, and increasing bystander behavior among college students who identified as women.

## Methods

### Recruitment and Study Design

A randomized controlled trial (RCT; ClinicalTrials.gov NCT03726437) was implemented at 3 universities in the southeastern United States. Study procedures were approved by the primary investigator’s university institutional review board. Eligible participants were (1) those who self-identified as women, (2) aged 18 to 20 years, (3) single, and (4) entering their first year of college. The eligibility criteria reflect the high risk of exposure to SV among first-year college women as well as the specific period of elevated risk for exposure to SV, which is the fall semester, also known as the *red zone* [34].

### Ethics Approval and Procedures

This study was approved by the Georgia State University institutional review board (H19033). Active recruitment began in October 2018 and ended in February 2019. We recruited a web-based sample of first-year college students who identified as women (n=881) using email contact lists provided by the registrar’s office of each university. An email was sent to potential participants containing a description of the study with a link to a web-based survey delivered via Qualtrics to complete an eligibility screener. If individuals met the eligibility criteria, they were then redirected to another web-based survey to complete the informed consent form and electronically provide their consent to participate. Potential participants were blinded to the study hypotheses and told that the purpose of the study was to “examine the effectiveness of a 3-hour web-based program for incoming female freshmen.”

Once they provided informed consent, participants were asked to complete a web-based registration form, where they provided their contact information, including their full name, email address, residential address, and mobile phone number. Next, participants were redirected to the baseline survey assessment. Participants received US $30 for completing the baseline survey. Once participants completed the baseline survey, they were directed to a web-based survey that collected data on their email address and institution, which was then used to randomize participants to 1 of the 2 study conditions. Stratified block randomization was implemented via REDCap (Research Electronic Data Capture; Vanderbilt University) to randomly assign participants to either RealConsent or to an attention-matched placebo condition called *Stress and Mood Management* [35]. We encouraged participants to complete their assigned program within a week by offering them a US $10 incentive for completing a brief acceptability survey after each program module. Participants were also asked to complete a follow-up survey 6 months after completing the baseline survey, for which they received US $50. Weekly email and SMS text reminders were sent to participants to complete program modules and surveys.

### Interventions

#### Experimental Intervention

**Figure 1:**
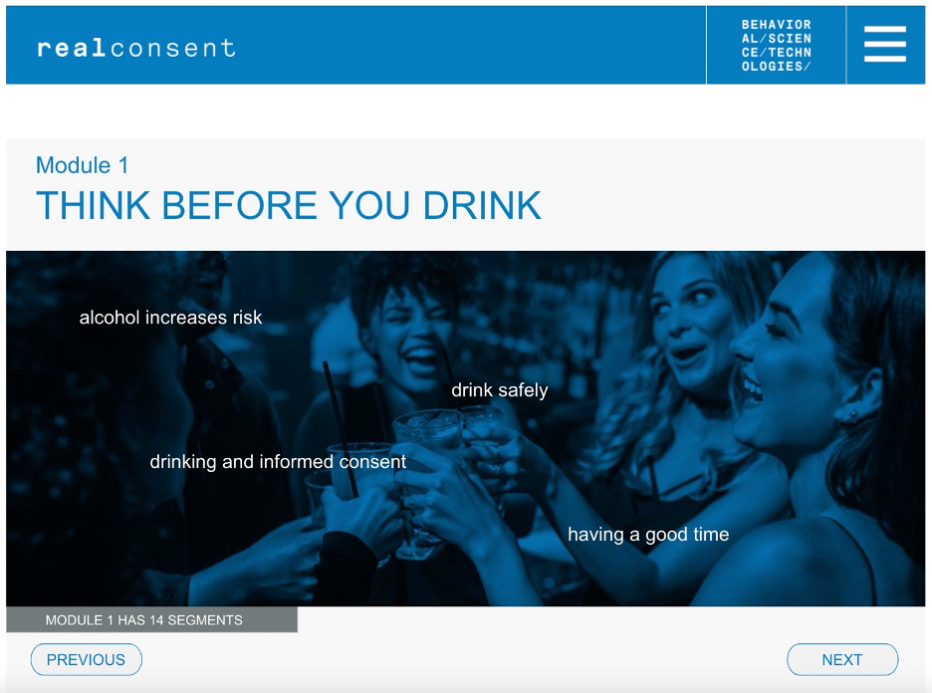
Screenshot of RealConsent module 1.

The RealConsent program (Figure 1) uses web-based and mobile technologies with the primary goal being to reduce the risk of exposure to SV among first-year college students who identify as women. RealConsent is grounded in the social cognitive theory (SCT) [36,37]. The SCT describes the multiple reciprocal influences on health behaviors, including individual experiences, beliefs, and environmental factors. According to the SCT, “knowledge of health risks and benefits of different health practices, perceived self-efficacy, outcome expectations about the expected costs and benefits, health goals people set for themselves, and perceived facilitators and social and structural impediments” can translate knowledge into effective health practices [37]. Building self-efficacy (confidence in one’s ability to perform a desired health behavior) and self-regulation (goal setting and planning) are key constructs of the SCT that guided the development of RealConsent.

In developing RealConsent, extensive formative research with the targeted population was conducted to assess the different contexts in which exposure to SV occurs, how men and women express sexual interest and consent, reasons for alcohol use, protective strategies to avoid SV, SV survivor blaming, barriers to bystander intervention, and stereotypical gender roles. The results were used to inform the content, messaging, language, and storylines for each segment and particularly for the serial drama titled *Squad*. Once the intervention module content was developed on paper, before production, an additional round of focus groups was conducted to assess the acceptability and relevance of the materials and scripts in terms of literacy, language, realism, presentation, and delivery.

The learning objectives of RealConsent were to (1) increase young women’s awareness of the risks of alcohol use, (2) enhance the skills necessary for engaging in alcohol-related protective behaviors, (3) raise awareness of dating-related risk factors for exposure to SV, and (4) teach effective prosocial bystander intervention and self-defense strategies. To achieve these objectives, RealConsent used several behavior change techniques [38] (eg, model or demonstrate behavior, provide information on consequences, and provide information on the link between behavior and health) to target SCT-related theoretical mediators [39] such as increasing knowledge of protective tactics while consuming alcohol; the role of alcohol use in SV and consent for sex; changing outcome expectancies toward consuming alcohol in risky contexts; increasing self-efficacy for bystander intervention, safe alcohol consumption, sexual communication, and protective sexual strategies; and increasing social learning strategies for bystander behavior, resistance behaviors, self-defense, assertive communication, and safe alcohol use. Refer to Multimedia Appendix 1 for the full module matrix with learning objectives, behavior change strategies used, theoretical mediators, and intended behavioral goal of each module.

RealConsent was delivered via a password-protected web portal that allowed participants to access the program either via the web or their mobile phones. The program contains four 45-minute program modules for a total duration of 3 hours. Each module includes interactivity, didactic activities, and entertainment-education media [40,41]. Entertainment-education is an effective health communication strategy that combines or embeds educational messages into entertainment programs to bring about social and behavior change [41]. RealConsent contains 8 mini-episodes of a serial drama titled *Squad* as its entertainment-education component. To ensure the relevance and quality of *Squad*, we worked with a professional scriptwriter so that our dialogue was realistic and entertaining. In addition, we contracted with a professional film and video company that had won several Emmy awards for their documentary films to film, direct, and edit our video segments. Consequently, the *Squad* serial drama garnered 4 Telly Awards. Telly Awards honor excellence in video and television across all platforms. The *Squad* episodes allow for the modeling of positive behaviors and for illustrating both positive and negative outcome expectations related to alcohol misuse and bystander intervention. RealConsent includes ethnically and racially diverse actors in its filmed segments and in images accompanying the didactic segments; in addition, it includes representation of same-sex relationships. RealConsent was programmed so that participants could not skip or click through segments within each module without viewing the entire segment. In addition, the program contained an administrative component that allowed study staff to track participants’ completion of the program.

#### Attention-Placebo Control Intervention

*Stress and Mood Management* is a web-based multimedia health promotion program developed by ISA Group and designed to help manage stress levels; prevent mood problems; and seek early identification of, and treatment for, depression and anxiety. Each of the 4 program modules is approximately 30 minutes long and involves videos as well as interactive and didactic activities. Thus, it approximates RealConsent in format and duration.

### Primary Outcome: Exposure to SV

Exposure to SV was assessed with the Revised Sexual Experiences Survey developed by Koss et al [42]. Behaviorally specific language was used to describe unwanted sexual experience outcomes and tactics. The types of unwanted sexual behavior assessed included sexual contact (eg, fondling) and attempted or completed penetration (oral, vaginal, or anal). The tactics included two forms of verbal coercion, including (1) telling lies, making verbal threats, making promises known to be untrue, or using verbal pressure; and (2) showing displeasure, criticizing, or getting angry, as well as incapacitation (ie, taking advantage when the participant was *too drunk or out of it* to stop what was happening), and two forms of physical force, including (1) threatening physical force and (2) the use of physical force. Participants were asked how often each sexual experience was obtained by each tactic, with response options ranging from 0 (*never*) to 3 (*≥3 times*). At baseline, *in the past 12 months* was used as the time reference, and at the 6-month follow-up, *since viewing the web-based program* was the time reference*.* Exposure to SV was analyzed in 2 ways as recommended by Davis et al [43] and takes into consideration the severity of the violence (eg, *fondling* vs *completed vaginal rape*) as well as the tactic used (eg, *alcohol incapacitated* vs *physical force*) in addition to the frequency. The combined outcomes separated tactics scoring method (COSTS) resulted in a continuous construct with a range of 0 to 63 where 6 severity ranks were used. For the rape outcomes that had the same tactic (eg, *attempted rape by force* and *completed rape by force*), each was given the same severity rank. Each severity score was multiplied by the frequency and then summed. The separated outcomes separated tactics scoring method (SOTS) also resulted in a continuous construct but with a range of 0 to 135 where each outcome by each tactic was ranked by severity from 1 (sexual contact with verbal coercion) to 9 (completed rape by force) and then multiplied by the frequency [43].

### Secondary Outcomes

#### Alcohol Protective Behaviors

Alcohol protective behaviors were assessed with 15 items from the Protective Behavioral Strategies Survey [44], with answer choices ranging on a 5-point scale from 1 (*always*) to 5 (*never*). Participants were asked whether, while using alcohol or *partying,* they engaged in alcohol-related protective behaviors (eg, *determine not to exceed a set number of drinks*, *avoid mixing different types of alcohol*, and *know where your drink has been at all times*). For this study, the scale showed adequate reliability (Cronbach α=.86). Items were summed for a total score for engaging in protective behaviors.

#### Dating Risk Behaviors

Dating risk behaviors were assessed using the Dating Behavior Survey, which consists of 15 items assessing the situational variables, including alcohol use, that have been found to be related to acquaintance rape [45]. Participants indicated how often they engaged in situational behaviors that would put them at risk (eg, on the first few dates...*I consume alcohol or drugs*, or *my partner and I do things that allow us to spend time alone together*). Answer choices ranged on a 5-point scale from 1 (*never*) to 5 (*always*). For this study, reliability was adequate (Cronbach α=.71). Responses were summed for a total score for risk-related dating behavior.

#### Alcohol Use

Alcohol use was assessed using several items from the Daily Drinking Questionnaire-Revised [46]. Participants were asked to report the number of times they consumed ≥4 alcoholic drinks in 1 sitting in the last 30 days (*binge drinking*), the number of drinks consumed as well as the number of hours they drank each day for a typical week (*average number of drinks per hour)*, and the number of drinks consumed on 1 occasion where they *drank the most* during the past 30 days (*heavy drinking)*.

#### Bystander Behavior

Bystander behavior was assessed using the 20-item Bystander Behavior Scale [47]. The items assessed whether participants engaged in bystander behaviors in the past 3 months and included items such as *If I saw someone taking a very intoxicated person up to their room, I said something and asked what the friend was doing*. Response options included how many times they intervened (eg, 1, 2, or ≥3) or *no opportunity*. For this study, reliability was adequate (Cronbach α=.84). Responses were summed for a total score for bystander behavior.

### Data Analysis

#### Statistical Power

The primary outcome was exposure to SV; the secondary outcomes were alcohol and dating protective behaviors, alcohol use behaviors, and bystander intervention. Sample size calculations for the primary outcome were estimated to guarantee that power would be at least 0.80 for the detection of a small-to-moderate effect size (Cohen *h*≥0.35). With 2 study groups, we estimated a needed sample size of at least 670; however, we factored in anticipated 20% attrition, which meant that we needed to enroll *at least* 750 participants (375 in each group) to increase power.

#### Data Analytic Strategy

Analyses were performed on prespecified hypotheses for the primary outcome variable of exposure to SV using an intent-to-treat protocol in which participants were analyzed according to their assigned study conditions [48]. Additional analyses were also performed on several secondary outcomes, including alcohol protective behaviors, dating risk behaviors, and alcohol misuse, using an intent-to-treat protocol. Statisticians were blinded to which group (*a* or *b*) was the experimental condition. An additional secondary outcome, bystander behavior, was also analyzed using a *dosage* protocol versus an intent-to-treat protocol. Participants who had completed 100% of the RealConsent program were compared with the control group participants plus those participants who had completed <100% of the program. Dosage was used to test for effects on bystander behavior because intervention content specific to bystander behavior was in the last module of the program.

Descriptive statistics were created for all study measures, with mean and SD for continuous variables and frequency distribution for categorical variables. Comparisons were conducted of baseline findings across study characteristics and outcomes to determine whether participants who completed the intervention were similar to those who did not.

A substantial number of participants were expected to report not having an experience of exposure to SV, to not have engaged in bystander behavior, and to not having previously consumed alcohol. To account for these zero occurrences, a comparison of the mean occurrence for each outcome across the baseline and 6-month follow-up time points was accomplished with a 2-stage modeling process using zero-inflated regression models. In the first stage, a logistic regression model was used to model occurrence or not for each outcome, and in the second stage, a Poisson or gamma regression model was used to model each outcome for those who had at least 1 occurrence. Exposure to SV and most of the alcohol consumption measurements were count outcomes and modeled with the Poisson distribution. The alcohol protective and dating risk behaviors scales were both continuous and reasonably symmetric. A multilevel model in the form of a general linear mixed model was used. The Bystander Behavior Scale was skewed and assessed with logistic regression; heavy drinking outcomes were continuous and skewed and were modeled with a gamma distribution.

Participants were assessed at baseline and at 6-month follow-up. Repeated measurements on each participant results in within-participant correlation. This was accounted for by estimating each zero-inflated model with population-averaged effects using a marginal model and generalized estimating equations. Each zero-inflated marginal model included fixed effects to control for study site, race, ethnicity, place of living, relationship status, sexual orientation, engagement in athletics, job status, ever drank alcohol, time, and study condition. A time × study condition interaction term was included in each model to assess and test for intervention effectiveness. The interaction term quantifies the relative change in the outcome over time across study conditions. Intervention effects were estimated with odds ratios for logistic models, incidence rate ratios (IRRs) for Poisson models, and regression coefficients for gamma models. SAS software (version 9.4; SAS Institute Inc) was used for all statistical analyses.

## Results

### Overview

**Figure 2:**
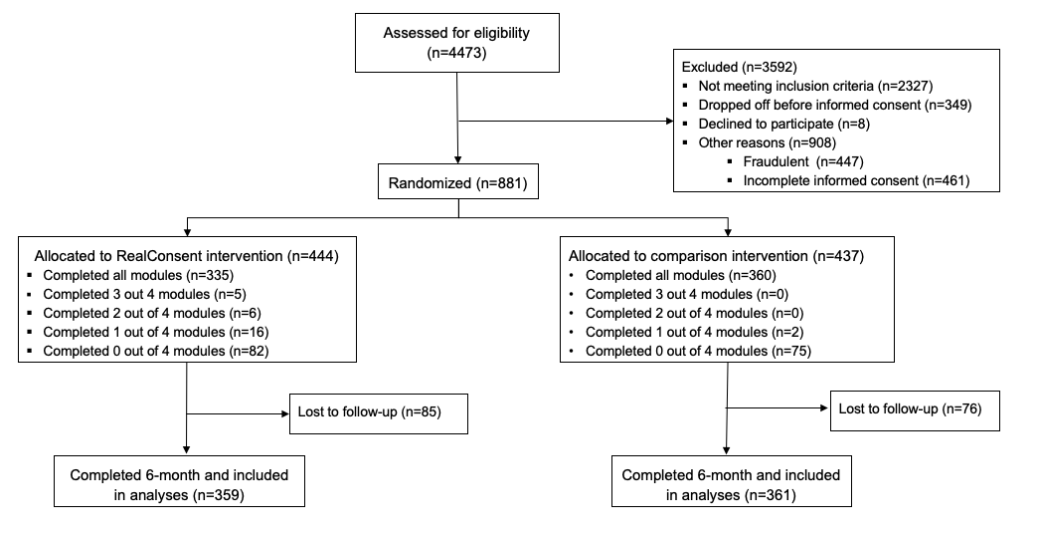
Study flowchart.

The recruitment process (Figure 2) resulted in 4473 first-year college students, who identified as women, who were screened for eligibility. Of these 4473 students, 2327 (52.02%) were not eligible, 349 (7.8%) did not undergo the informed consent process, 8 (0.18%) declined to participate, and 908 (20.3%) did not enroll for other reasons, whereas 881 (19.7%) consented, completed baseline assessment, and were then randomized. At 6 months, 161 (18.3%) of the 881 participants were lost to follow-up. The chi-square results indicated that there was not differential attrition: 85 (19.1%) of the 444 participants in the RealConsent condition were lost to follow-up versus 76 (17.4%) of the 437 participants in the placebo comparison condition (*P*=.83).

### Participant Characteristics

Being aged 18 to 20 years was an eligibility criterion; thus, the sample mirrored this age range. Most of the participants (642/881, 72.9%) were aged 18 years, followed by those aged 19 years (230/881, 26.1%) and 20 years (9/881, 1%). The racial breakdown of participants was as follows: American Indian or Alaska Native (6/881, 0.7%), Asian (176/881, 20%), Black or African American (213/881, 24.2%), Native Hawaiian or Pacific Islander (3/881, 0.3%), White (394/881, 44.7%), biracial or multiracial (76/881, 8.6%), and other (13/881, 1.5%). Hispanic or Latinx participants constituted 12.3% (108/881) of the sample. In terms of gender identity, most of the participants (873/881, 99.1%) identified as woman, and 0.9% (8/881) identified as nonconforming or nonbinary. Regarding sexual orientation, most of the participants (724/881, 82.2%) identified as heterosexual, 11.4% (100/881) identified as bisexual, 2.5% (22/881) identified as gay or lesbian, 1.5% (13/881) as queer, and 2.5% (22/881) as other. Most of the participants were full-time students (879/881, 99.8%), single (845/881, 95.9%), and lived in campus dorms or housing (614/881, 69.7%). A small number of the participants were members of athletic teams (115/881, 13.1%). Overall, the prevalence of exposure to SV before starting college was 27.4% (241/881). Table 1 provides data on the breakdown of sociodemographic variables and outcome variables by study condition.

**Table 1:** Baseline characteristics stratified by RealConsent and control group participants (n=881).

Characteristics			*RealConsent* (n=444)	Control condition (n=437)
**Sociodemographics, n (%)**				
	Engages in athletics		59 (13.3)	56 (12.8)
	Ever drank alcohol		307 (69.1)	312 (71.4)
	Ever on a date		324 (73)	301 (68.9)
	Has a job		162 (36.5)	138 (31.6)
	**Race and ethnicity**			
		American Indian or Alaska Native	2 (0.5)	4 (0.9)
		Asian	87 (19.6)	89 (20.4)
		Black or African American	104 (23.5)	109 (24.9)
		Hispanic or Latinx	51 (11.5)	57 (13)
		Native Hawaiian or Pacific Islander	2 (0.5)	1 (0.2)
		White	198 (44.6)	196 (44.9)
		Biracial or multiracial	42 (9.5)	34 (7.8)
	**Place of living**			
		On campus dorm	353 (79.5)	364 (83.3)
		Other	91 (20.5)	73 (16.7)
	**Sexual orientation**			
		Heterosexual	363 (81.8)	361 (82.6)
		Other	81 (18.2)	76 (17.4)
	**Relationship status**			
		Single	425 (95.7)	420 (96.1)
		In a relationship	19 (4.3)	17 (3.9)
	**University**			
		Private	90 (20.3)	90 (20.6)
		Public urban	200 (45)	199 (45.5)
		Suburban	154 (34.7)	148 (33.9)
**Primary outcomes, mean (SD)**				
	Exposure to SV^a^ (SOTS^b^)		5.6 (15.70)	5.7 (17.10)
	Exposure to SV (COSTS^c^)		3.7 (8.90)	3.8 (9.80)
**Secondary outcomes, mean (SD)**				
	Alcohol protective behaviors		53.53 (12.17)	53.97 (11.35)
	Dating risk behaviors		35.16 (5.10)	35.08 (4.80)
	Binge drinking		1.71 (2.53)	1.25 (1.80)
	Bystander behavior		7.0 (8.90)	6.5 (9.00)

^a^SV: sexual violence.

^b^SOTS: separated outcomes separated tactics scoring method.

^c^COSTS: combined outcomes separated tactics scoring method.

### Completers Versus Noncompleters

Analyses were completed to assess the differences in study covariates and outcomes at baseline between those who completed the 6-month follow-up assessment and those who were lost to follow-up. As shown in Table 2, there was a higher percentage of dropouts versus completers (28/161, 17.4% vs 80/720, 11.1%, respectively) who identified as Hispanic (*P*=.03). On average, completers had lower levels of binge drinking and dating risk behaviors. Completers and noncompleters did not differ on any other covariates or outcomes.

**Table 2:** Comparisons on baseline covariates and study outcomes for noncompleters versus completers (n=881).

Covariate		Noncompleters (n=161)	Completers (n=720)	*P v*alue	
**Engages in athletics^a^, n (%)**					.44
	Yes	24 (14.9)	91 (12.6)		
	No	137 (85.1)	629 (87.4)		
**Ever drank alcohol^a^, n (%)**					.19
	Yes	120 (74.5)	499 (69.3)		
	No	41 (25.5)	221 (30.7)		
**Ever on a date^a^, n (%)**					.06
	Yes	124 (77)	502 (69.7)		
	No	37 (23)	218 (30.3)		
**Has a job^a^, n (%)**					.97
	Yes	55 (34.2)	245 (34)		
	No	106 (65.8)	475 (66)		
**Hispanic^a^, n (%)**					.03
	Yes	28 (17.4)	80 (11.1)		
	No	133 (82.6)	640 (88.9)		
**Place of living^a^, n (%)**					.65
	On campus	129 (80.1)	588 (81.7)		
	Off campus	32 (19.9)	132 (18.3)		
**Race^a^, n (%)**					.09
	Black or African American	36 (22.4)	177 (24.6)		
	White	84 (52.2)	309 (43)		
	Other	41 (25.5)	233 (32.4)		
**Relationship status^a^, n (%)**					.53
	Single	153 (95)	692 (96.1)		
	In a relationship	8 (5)	28 (3.9)		
**Sexual orientation^a^, n (%)**					.23
	Heterosexual	127 (78.8)	597 (82.9)		
	Nonheterosexual	34 (21.1)	123 (17.1)		
**University^a^, n (%)**					.66
	Private	37 (23)	143 (23)		
	Public urban	72 (44.7)	327 (45.5)		
	Public suburban	52 (32.3)	249 (34.6)		
**Primary outcomes, mean (SD)**					
	SV^b^: SOTS^c,d^	7.3 (18.9)	5.3 (15.7)	.16	
	SV: COSTS^d,e^	4.7 (10.6)	3.5 (9.1)	.16	
**Secondary outcomes, mean (SD)**					
	Alcohol protective behaviors^d^	53.7 (11.0)	53.8 (11.9)	.95	
	Dating risk behaviors^d^	36.3 (5.2)	34.8 (4.9)	.005	
	Binge drinking^d^	2.2 (2.7)	1.3 (2.0)	<.001	
	Bystander behavior^d^	8.1 (9.4)	6.6 (8.9)	.06	

^a^Chi-square test of independence.

^b^SV: sexual violence.

^c^SOTS: separated outcomes separated tactics scoring method.

^d^Independent samples 2-tailed *t* test.

^e^COSTS: combined outcomes separated tactics scoring method.

### Primary Outcome (Exposure to SV): SOTS and COSTS Outcomes

The sexual experience survey measures frequency and severity of exposure to SV in the form of behavioral counts. Thus, to model these counting processes, a Poisson regression framework was used for the SOTS and COSTS outcomes. Of the 881 participants, 640 (72.6%) had a zero baseline SOTS and COSTS score. To account for repeated measurements taken over 2 time points and the excess zeros, multilevel zero-inflated Poisson regression models were used. With multilevel zero-inflated Poisson models, the outcome was modeled as a mixture of 2 distributions. First, a binomial distribution was used to fit a logistic regression model to a dichotomy: the occurrence of exposure to SV (score>0) versus no exposure to SV (score=0). For this logistic regression, only 1 model was run because the dichotomy distributions for SOTS and COSTS are equivalent. Second, a Poisson regression model with overdispersion was used to model participants who had some occurrence of exposure to SV (score>0). A random effect for participant was included in each model to account for the correlated repeated measurements resulting from baseline and follow-up measurements. The results are presented in Table 3.

**Table 3:** Multilevel modeling results for exposure to sexual violence (SV) outcome (separated outcomes separated tactics scoring method [SOTS] and combined outcomes separated tactics scoring method [COSTS]).

Exposure to SV outcome				*P* value
**SOTS and COSTS: zero model^a^, adjusted odds ratio^b^ (95% CI)**				
	RealConsent	0.77 (0.47-1.28)	.31	
	Control	Reference	N/A^c^	
**SOTS: Poisson model^a^,incidence rate ratio^b^ (95% CI)**				
	RealConsent	0.48 (0.33-0.69)	.002	
	Control	Reference	N/A	
**COSTS: Poisson model^a^, incidence rate ratio^b^ (95% CI)**				
	RealConsent	0.56 (0.40-0.78)	.001	
	Control	Reference	N/A	

^a^All models adjusted for time, race, ethnicity, place of living, relationship status, sexual orientation, engagement in athletics, job status, ever drank alcohol, and ever on a date.

^b^Time by study condition interaction effect estimates displayed.

^c^N/A: not applicable.

As shown in Table 3, for the zero-inflated SOTS and COSTS model for exposure to SV, controlling for other covariates, although the adjusted odds ratio (AOR) was protective (ie, less likely to experience SV over time), this result was not significant (AOR 0.77, 95% CI 0.47-1.28; *P*=.32). For the Poisson regression model with overdispersion, controlling for other covariates, participants in the RealConsent group who had experienced some exposure to SV were found to have a significant decrease in levels of exposure to SV compared with those in the control condition (adjusted IRR estimate 0.48, 95% CI 0.33-0.69; *P*<.001).

For COSTS, similarly, for the Poisson regression model with overdispersion, controlling for other covariates, participants in the RealConsent group who had experienced some exposure to SV were found to have a significant decrease in levels of exposure to SV compared with those in the control condition (adjusted IRR estimate 0.56, 95% CI 0.40-0.78; *P*=.001).

### Secondary Outcomes

#### Alcohol Protective Behaviors

The effect of RealConsent on alcohol protective behaviors was modeled with a general linear mixed model. The time × study condition effect was significant (AOR 1.17, 95% CI 0.12-2.22; *P*=.03). Participants in the RealConsent group were more likely to engage in alcohol-related protective behaviors than those in the placebo control condition.

#### Dating Risk Behaviors

The effect of RealConsent on dating risk behaviors was modeled with a general linear mixed model. Although the effect was in the hypothesized direction, the time × study condition effect was not significant (AOR −0.50, 95% CI −1.27 to 0.27; *P*=.20).

#### Alcohol Use

We examined the effects of RealConsent on several alcohol outcome variables. As shown in Table 4, no significant results were found for number of alcohol drinks per occasion or average number of alcoholic drinks per hour. Significant results were found for binge drinking: for the Poisson regression model with overdispersion, controlling for covariates, participants in the RealConsent group had lower rates of binge drinking than those in the control condition (adjusted IRR 0.81, 95% CI 0.67-0.97; *P*=.02).

**Table 4:** Multilevel modeling results for alcohol use outcomes.

Outcome				*P* value
**Number of drinks per occasion during the past 30 days: Poisson model^a^, adjusted incidence rate ratio^b^ (95% CI)**				
	RealConsent	1.05 (0.92 to 1.20)	.43	
	Control	Reference	N/A^c^	
**Binge drinking (ie, ≥4 drinks in 1 setting): Poisson model^a^, adjusted incidence rate ratio^b^ (95% CI)**				
	RealConsent	0.81 (0.67 to 0.97)	.02	
	Control	Reference	N/A	
**Heavy drinking measured as average number of drinks per hour: gamma model^a^, parameter estimate^b^ (95% CI)**				
	RealConsent	−0.05 (−0.22 to 0.11)	.54	
	Control	Reference	N/A	

^a^All models adjusted for time, race, ethnicity, place of living, relationship status, sexual orientation, engagement in athletics, job status, ever drank alcohol, and ever on a date.

^b^Time by study condition interaction effect estimates displayed.

^c^N/A: not applicable.

#### Bystander Behavior

To test whether RealConsent was effective in increasing bystander behavior, we tested the model using *dosage* as the independent variable (because module 4, the last module, was specific to bystander intervention). The dosage groups were as follows: (1) the RealConsent group participants at 6-month follow-up who had completed 4 modules (305/444,68.7%) versus (2) the RealConsent group participants who had not completed all 4 modules (54/444, 12.2%) plus the control group participants (361/437, 82.6%). The distribution at baseline for this continuous variable included 33.5% (241/720) of the participants who did not exhibit bystander behavior (score=0). A multilevel logistic regression model was developed. This modeling approach accounts for the within-participant correlation resulting from repeated measurements taken over 2 time points. The outcome was occurrence of bystander behavior (score>0) versus no bystander behavior (score=0). A random effect for participant was included in the model to account for the correlated repeated measurements resulting from baseline and follow-up measurements. Controlling for covariates, participants in the RealConsent group who had 100% dosage were found to have increased odds of engaging in any bystander behavior compared with those who had <100% dosage plus the control group (AOR estimate 1.72, 95% CI 1.17-2.55; *P*=.006).

## Discussion

### Principal Findings

This study is the first comprehensive SV risk reduction program specific to women that incorporates entertainment-education media into web-based and mobile technologies, is theoretically and empirically informed, embodies proven behavior change techniques, includes alcohol education as a central component, and integrates bystander education as well as self-defense training—all factors associated with SV risk reduction. The results from this RCT demonstrate significant changes in both primary and secondary outcomes among a racially diverse sample of first-year college students who identified as women. Among participants who had experienced any SV, RealConsent participants reported less exposure to SV (primary outcome) than control group participants (*P*<.001). This result suggests that RealConsent is effective for participants who are most at risk. Although we did not find significant results for our zero-inflated logistic model, which compared no exposure to SV to any exposure to SV by condition, we can speculate possible reasons for this null finding. First, because of RealConsent’s central focus on alcohol misuse and highlighting the heightened risks for alcohol-related SV, it may be that the content resonated more with participants who had been previously exposed to SV because research shows that alcohol misuse can be a consequence of exposure to SV [4,49,50]. Another potential explanation relates to external events. Since the passing of the Campus Sexual Violence Elimination Act in 2013, institutions of higher learning (those that participate in federal student aid programs) are required to provide campus community-wide prevention educational programming. The college students who identified as women in our sample most likely underwent their respective colleges’ required SV prevention programming, which incoming first-year students are mandated to complete the summer before the semester begins. In addition, campus administrative offices such as residence life, student health services, and student affairs may have also implemented additional trainings and awareness events throughout the fall semester during the high-risk period known as the *red zone* [34,51,52]. The *red zone* describes the heightened risk of exposure to SV for first-year college students who identified as women during their fall semester [34,51,52]. As the study participants were recruited from 3 separate universities, it is likely that students were exposed to other SV prevention messaging during the study implementation period [34,52]. It is plausible that these external events affected overall exposure to SV incidence at follow-up, resulting in inadequate statistical power and contributing to the null findings. Moreover, research has shown that overall rates of exposure to SV drop after the first semester’s *red zone* period has elapsed. Thus, a naturally occurring drop in SV rates overall could have contributed to the null findings. A final potential explanation is contamination, whereby some of the participants in the experimental group may have discussed or even shared some of the information learned with friends who were in the attention-matched placebo group. Contamination generally biases the estimated treatment effect toward the null [53]. Trials of educational prevention interventions are particularly vulnerable to such bias [53]. In this trial, participants in the attention-matched placebo arm may have been exposed to RealConsent messages indirectly through participants in the RealConsent condition interacting with them and possibly sharing information, promoting new social norms, or even encouraging and modeling behavior change. As randomization was at the individual level, and 69.7% (614/881) of our sample reported living in the dorms, that is, in close proximity to each other, and the participants in this trial were all first-year students, contamination for this null effect could be a possible explanation.

With a preponderance of evidence to support the link between alcohol misuse and exposure to SV [15,30,31,49-51,54] and with research showing that alcohol consumption that results in incapacitation or blackouts is common on college campuses [55,56], it is critical to approach SV risk reduction by targeting both alcohol misuse and alcohol-related protective behaviors such as *know where your drink has been at all times* and *alternate alcoholic and nonalcoholic drinks*. RealConsent showed a significant increase in alcohol-related protective behaviors (*P*=.03) and was protective against binge drinking (*P*=.003) (secondary outcomes). Further research that examines whether these secondary outcomes act as mediators of RealConsent’s effect on exposure to SV outcomes is warranted.

We also observed significant effects (*P*=.006) for 1 other secondary outcome: bystander behavior. Although there are various effective bystander intervention programs [57-60], to our knowledge, only 1 other program has been *specific to women* (*The Women’s Program* [61]); however, bystander behaviors were not assessed in the study, and it is unclear whether the program affected bystander behavior. One critique of SV risk reduction targeting women has been that effects are limited to only those women who undergo the program and that a burden is placed on women as solely responsible for avoidance of exposure to SV [62]. It is plausible that by incorporating bystander education into our SV risk reduction program, this burden as well as defensiveness were reduced, and women felt empowered. Future research that examines these potential mechanisms is warranted.

The range of effects observed in this RCT could be explained by several factors. First, in developing RealConsent, we engaged in extensive formative research to ensure acceptability and relevance. These formative research steps have been highlighted as being critical for developing effective interventions [63] and were also used to develop the men's version of RealConsent [64]. The acceptability of an intervention is necessary but perhaps not sufficient for effectiveness; however, if an intervention is considered acceptable, then the target audience is more likely to benefit from improved outcomes [65].

In addition, intervention implementation, delivery, and mode of delivery are relevant to acceptability. Delivery and the mode of delivery can include program format and content, which may considerably affect an intervention [2]. Mode of delivery may be especially relevant for members of Generation Z (those born between 1996 and 2015 and our targeted population), for whom social media, constant connectivity, and on-demand entertainment and communication were the norm growing up [66]. With the ubiquity and popularity of streaming services (eg, Netflix, Amazon Prime, and Hulu) to access entertainment, especially for members of Generation Z, RealConsent’s mode of delivery via web-based or mobile platforms may have resonated and been more acceptable versus an in-person didactic mode and may have contributed to the effects observed. Equally important to the mode of delivery, however, is the production quality of the entertainment-education media within a program. With a vast array of high-quality and high-production content available and regularly viewed by our targeted population, it is important to consider whether an entertainment-education effort can successfully garner participant interest in an environment full of competing entertainment messages. Although participants agreed to complete the program modules as part of this RCT, completion was not mandatory, and all participants had the option to drop out, withdraw, or, if so inclined, simply not view the content while it was running. In viewing RealConsent completion rates, our results show that 75.5% (335/444) of the participants completed all 4 modules of the program, which can be viewed as a high percentage of completion for a web-based program [67] and which suggests perceived relevance and quality. Further research regarding the acceptability of RealConsent and its relevance and perceived quality in terms of entertainment value is warranted.

Another aspect of program format related directly to observed effects is duration. As is the case with efficacious SV risk reduction programs such as the Enhanced Assess, Acknowledge, Act program developed by Senn et al [23] and the self-defense classes for college women analyzed by Hollander [32], longer duration has been effective in affecting behavior and improving attitudes, whereas programs with a brief duration (eg, a lecture) may be more impactful at reducing rape myth acceptance [2]. One exception is the web-based SV and alcohol use program developed by Gilmore et al [27], which targeted college women aged 18 to 20 years who engage in heavy episodic drinking (eg, consuming ≥4 alcoholic beverages at least twice within the past month). Although shorter in duration than the program developed by Senn et al [23] (12 hours) or the self-defense classes for college women (45 hours) [32], RealConsent’s 3-hour duration produced efficacious results at 6 months for women most at risk and for multiple secondary outcomes. Further research is warranted to examine whether the observed effects would be maintained at 1 year.

Another program aspect that may be related to efficacy is the use of a theoretical framework and behavior change techniques to inform and guide content and activities. Research has overwhelmingly shown that health behavior theories contribute greatly to our understanding of behavior and behavior change [36]. In addition, a systematic review and meta-analysis of web-based eHealth behavioral interventions found that the use of health behavior theory and incorporation of more behavior change techniques were both associated with greater effect sizes [68], suggesting that, in general, behavioral interventions guided by health behavior theory are more efficacious than nontheory-based interventions. The SCT guided the development of RealConsent content. We also incorporated multiple proven behavior change techniques [38], such as modeling positive behaviors, providing information on consequences of behavior, providing information on the link between behavior and health outcomes, identifying barriers, and providing feedback on performance. This comprehensive approach to intervention development may have improved the ability of the RealConsent intervention to achieve significant effects on several behavioral outcomes. Further research that examines whether the significant effects observed are direct effects or indirect effects through the theoretical mediators is warranted and will reveal RealConsent’s mechanism of action. Such information can be used for future behavioral interventions targeting similar outcomes. Finally, single-sex program formats for female participants have also been found to be more effective than mixed-sex programming at improving a host of risk factors for exposure to SV [2] and may have contributed to the observed significant findings.

### Limitations and Strengths

Our trial included several limitations. First, our trial was conducted with first-year college students who identified as women, recruited from 3 universities located in the southeastern United States. Future research should test RealConsent among college women at universities located in other geographical areas of the United States. Second, although it was not extreme, we experienced some loss to follow-up in terms of completion of the respective web-based programs and follow-up survey. It is unclear what the potential reasons were for this loss to follow-up; however, previous research has shown that attrition in web-based trials may be higher than that in in-person trials [67,69,70]. Nevertheless, our observed attrition rates (overall 161/881, 18.3%) are lower than (eg, 40% [71]), or in line with (eg, 18% [72]), other *in-person* SV risk reduction trials with similar follow-up periods. Third, although the RCT design controls for many threats to internal validity, this trial was implemented in the field as opposed to a laboratory setting, where it was impossible to control for all external events. Nonetheless, there are several strengths. RealConsent was developed with extensive formative research with the targeted population, and its content is theoretically informed. The RCT was implemented with a large racially diverse sample of college students who identified as women; in addition, participants were masked to study hypotheses and biostatisticians were blinded to group condition, all of which represent significant methodological strengths.

### Conclusions

This RCT showed that a 3-hour comprehensive web-based or mobile-based program tailored to first-year college women was efficacious in substantially reducing the occurrence of exposure to SV among those at higher risk because of previous exposure to SV, reducing binge drinking, increasing alcohol-related protective behaviors, and increasing bystander behavior. RealConsent can be easily disseminated owing to its web-based and mobile technology and holds potential to reach large numbers of college women and perhaps reduce overall incidence of exposure to SV.

## Appendix

Multimedia Appendix 1 RealConsent modules matrix.

Multimedia Appendix 2 CONSORT-eHEALTH checklist (V 1.6.1).

## Abbreviations

AOR: adjusted odds ratio
COSTS: combined outcomes separated tactics scoring method
IRR: incidence rate ratio
REDCap: Research Electronic Data Capture
RCT: randomized controlled trial
SCT: social cognitive theory
SOTS: separated outcomes separated tactics scoring method
SV: sexual violence
