# Supplementary material for: A Web-Based Sexual Violence, Alcohol Misuse, and Bystander Intervention Program for College Women (RealConsent): Randomized Controlled Trial
Source: J Med Internet Res. 2023 Jun 21;25:e43740. doi: 10.2196/43740 (PMC10337467; doi:10.2196/43740)
Supplement: Multimedia Appendix 1 [file jmir_v25i1e43740_app1.pdf]

Multimedia Appendix of RealConsent Modules Matrix.

Modules*	Learning Objectives	Module Segments	Theoretical Mediators	Behavioral Goal
Think Before You Drink	• Increase understanding of how alcohol (and especially heavy alcohol use) is a situational risk factor for sexual assault<br>• Learn to identify situations that involve alcohol consumption and require help from network members<br>• Increase understanding of how alcohol alters men and women's behavior and reduces women's perceptions of risk<br>• Learn how alcohol affects cognitive abilities; learn how alcohol can influence informed consent<br>• Learn how to identify risky situations for a sexual assault and to identify good decision-making strategies when at a party or bar and how to cope with peer pressure to consume alcohol	Module Content:<br>1. Learning Objectives<br>2. Video with sexual assault educator<br>3. Serial Drama Squad episode 1 (video)<br>4. Video on alcohol and sexual assault<br>5. Didactic quizzes<br>6. Video on alcohol abuse<br>7. Serial Drama Squad episode 2<br>8. Group of young women debriefing on Squad episodes (video)<br>9. Video with sexual assault health educator<br>10. Video on alcohol abuse<br>11. Video on tips for going out safely<br>14. Module recap	• Knowledge on role of alcohol use in victimization and self-defense<br>• Personal and social outcome expectancies for alcohol consumption<br>• Self-efficacy related to decreased alcohol use in risky contexts<br>• Social learning of responsible alcohol use	• Decrease alcohol use in risky contexts
Let's Get Consensual	• Increase knowledge of effective consent – fully conscious, equal ability to act, sincere, clearly communicated<br>• Increase knowledge of the risk factors for rape and sexual assault victimization<br>• Understand the negative consequences of rape and sexual assault upon both women and men	Module Content:<br>1. Learning Objectives<br>2. Video with sexual assault educator<br>3. Serial Drama Squad episode 3<br>4. Video on sexual consent<br>5. Video on 4 elements of consent<br>6. Didactic content on rape myths<br>7. Serial Drama Squad episode 4	• Knowledge of informed consent<br>• Outcome expectancies of engaging in risky dating behaviors<br>• Positive outcome expectancies for protective dating behaviors	• Increase communication about sexual consent

	• Debunk rape myths and enhance understanding of contributors to rape and sexual assault<br>• Increase knowledge of national and local statistics and information on rape and sexual assault<br>• Increase empathy for rape victims	8. Group of young women debriefing on Squad episodes (video)<br>9. Video on accounts of sexual account<br>10. Video with sexual assault educator<br>11. Module recap	• Social learning of communication about sex<br>• Knowledge about assertive communication<br>• Self-efficacy to communicate assertively<br>• Self-efficacy to communicate sexual consent	
Can We Just Talk?	• Identify barriers to effective communication with a male partner<br>• Learn strategies for overcoming communication barriers and enhance communication skills<br>• Build self-efficacy to engage in communication in general and in situations involving bystander intervention<br>• Emphasize consequences of making good choices surrounding bystander intervention and sexual behavior<br>• Learn assertive communication for intervening and for articulating sexual boundaries, wants, and need	Module content:<br>1. Learning Objectives<br>2. Video with sexual assault health educator<br>3. Serial drama Squad video episode 5<br>4. Video on dating, communication, and risk-reducing strategies<br>5. Serial drama Squad video episode 6<br>6. Group of young women debriefing on Squad episodes (video)<br>7. Short video with static images on communicating sexual consent and refusing sex<br>8. Video with sexual assault health educator<br>9. Interactive quiz<br>10. Video with sexual assault health educators<br>11. Module recap	• Increase knowledge about assertive communication and bystander intervention<br>• Social learning of communication about sex<br>• Self-efficacy to communicate about sex	• Increase sexual communication

Circle of Defense	• Identify barriers to creating a supportive network that can be tapped into for help in a risky situation<br>• Learn strategies and techniques to resist unwanted sexual activities and for effective self-defense<br>• Build self-efficacy to engage in resistance strategies and self-defense techniques<br>• Emphasize positive consequences of using Circle of 6 app and encouraging friends to create supportive networks	Module content:<br>1. Learning Objectives<br>2. Video on bystander intervention and protective behaviors<br>3. Video with sexual assault health educators<br>4. Serial drama Squad episode 7<br>5. Interactive quiz on bystander intervention and protective behaviors<br>6. Video reinforcing positive consequences of bystander intervention<br>7. Serial drama Squad episode 8<br>8. Group of young women debriefing on Squad episodes (video)<br>9. Video on bystander intervention strategies<br>10. Video with sexual assault health educators<br>11. Self-defense tutorial<br>12. Interactive module on making a plan ahead of time to ensure safety<br>13. Module recap	• Knowledge of bystander intervention and protective behaviors to prevent SV and self-defense<br>• Self-efficacy to engage in protective behaviors and encouraging friends to do the same<br>• Positive outcome expectancies for self-protective behaviors and encouraging friends to do the same	• Increase bystander intervention<br>• Engaging in self-protective behaviors<br>• Encouraging friends to intervene and engage in self-protective behaviors

Module content is presented in order of which they appear in the RealConsent program.
