# Supplementary material for: A Web-Based Sexual Violence, Alcohol Misuse, and Bystander Intervention Program for College Women (RealConsent): Randomized Controlled Trial
Source: J Med Internet Res. 2023 Jun 21;25:e43740. doi: 10.2196/43740 (PMC10337467; doi:10.2196/43740)
Supplement: Multimedia Appendix 2 [file jmir_v25i1e43740_app2.pdf]

CONSORT-EHEALTH (V 1.6.1) - Submission/Publication Form

The CONSORT-EHEALTH checklist is intended for authors of randomized trials evaluating web-based and Internet-based applications/interventions, including mobile interventions, electronic games (incl multiplayer games), social media, certain telehealth applications, and other interactive and/or networked electronic applications. Some of the items (e.g. all subitems under item 5 - description of the intervention) may also be applicable for other study designs.

The goal of the CONSORT EHEALTH checklist and guideline is to be
a) a guide for reporting for authors of RCTs,
b) to form a basis for appraisal of an ehealth trial (in terms of validity)

CONSORT-EHEALTH items/subitems are MANDATORY reporting items for studies published in the Journal of Medical Internet Research and other journals / scientific societies endorsing the checklist.

Items numbered 1., 2., 3., 4a., 4b etc are original CONSORT or CONSORT-NPT (non-pharmacologic treatment) items.
Items with Roman numerals (i., ii, iii, iv etc.) are CONSORT-EHEALTH extensions/clarifications.

As the CONSORT-EHEALTH checklist is still considered in a formative stage, we would ask that you also RATE ON A SCALE OF 1-5 how important/useful you feel each item is FOR THE PURPOSE OF THE CHECKLIST and reporting guideline (optional).

Mandatory reporting items are marked with a red *.
In the textboxes, either copy & paste the relevant sections from your manuscript into this form - please include any quotes from your manuscript in QUOTATION MARKS,
or answer directly by providing additional information not in the manuscript, or elaborating on why the item was not relevant for this study.

YOUR ANSWERS WILL BE PUBLISHED AS A SUPPLEMENTARY FILE TO YOUR PUBLICATION IN JMIR AND ARE CONSIDERED PART OF YOUR PUBLICATION (IF ACCEPTED).
Please fill in these questions diligently. Information will not be copyedited, so please use proper spelling and grammar, use correct capitalization, and avoid abbreviations.

DO NOT FORGET TO SAVE AS PDF _AND_ CLICK THE SUBMIT BUTTON SO YOUR ANSWERS ARE IN OUR DATABASE !!!

Citation Suggestion (if you append the pdf as Appendix we suggest to cite this paper in the caption):
Eysenbach G, CONSORT-EHEALTH Group

CONSORT-EHEALTH: Improving and Standardizing Evaluation Reports of Web-based and Mobile Health Interventions
J Med Internet Res 2011;13(4):e126
URL: http://www.jmir.org/2011/4/e126/
doi: 10.2196/jmir.1923
PMID: 22209829

lsalazar1@gsu.edu Switch account

Not shared

Draft saved

* Indicates required question

Your name *

First Last

Laura F. Salazar

Primary Affiliation (short), City, Country *

University of Toronto, Toronto, Canada

Georgia State University, Atlanta, Georgia, USA

Your e-mail address *

abc@gmail.com

lsalzar1@gsu.edu

Title of your manuscript *

Provide the (draft) title of your manuscript.

A Web-based Sexual Assault, Alcohol Misuse and Bystander Intervention Program for College Women (RealConsent): Randomized Controlled Trial

Name of your App/Software/Intervention *

If there is a short and a long/alternate name, write the short name first and add the long name in brackets.

RealConsent

Evaluated Version (if any)

e.g. "V1", "Release 2017-03-01", "Version 2.0.27913"

Version for Women

Language(s) *

What language is the intervention/app in? If multiple languages are available, separate by comma (e.g. "English, French")

English

URL of your Intervention Website or App

e.g. a direct link to the mobile app on app in appstore (itunes, Google Play), or URL of the website. If the intervention is a DVD or hardware, you can also link to an Amazon page.

https://realconsent.com/

URL of an image/screenshot (optional)

Your answer

Accessibility *

Can an enduser access the intervention presently?

- ○ access is free and open
- ◉ access only for special usergroups, not open
- ○ access is open to everyone, but requires payment/subscription/in-app purchases
- ○ app/intervention no longer accessible
- ○ Other:

Primary Medical Indication/Disease/Condition *

e.g. "Stress", "Diabetes", or define the target group in brackets after the condition, e.g. "Autism (Parents of children with)", "Alzheimers (Informal Caregivers of)"

N/A (Women College Students)

Primary Outcomes measured in trial *

comma-separated list of primary outcomes reported in the trial

Sexual Violence Victimization

Secondary/other outcomes

Are there any other outcomes the intervention is expected to affect?

Alcohol Misuse, Alcohol Protective Behaviors, Dating Risk Behaviors, Bystander Behavior

Recommended "Dose" *

What do the instructions for users say on how often the app should be used?

- ○ Approximately Daily
- ○ Approximately Weekly
- ○ Approximately Monthly
- ○ Approximately Yearly
- ○ "as needed"
- ◉ Other: One-time use web-based program

Approx. Percentage of Users (starters) still using the app as recommended after 3 months *

- ○ unknown / not evaluated
- ○ 0-10%
- ○ 11-20%
- ○ 21-30%
- ○ 31-40%
- ○ 41-50%
- ○ 51-60%
- ○ 61-70%
- ○ 71%-80%
- ○ 81-90%
- ○ 91-100%
- ◉ Other: Program is one-time use

Overall, was the app/intervention effective? *

- ○ yes: all primary outcomes were significantly better in intervention group vs control
- ○ partly: SOME primary outcomes were significantly better in intervention group vs control
- ○ no statistically significant difference between control and intervention
- ○ potentially harmful: control was significantly better than intervention in one or more outcomes
- ○ inconclusive: more research is needed
- ◉ Other: Primary outcome was significantly better in intervention group who ha

Article Preparation Status/Stage *

At which stage in your article preparation are you currently (at the time you fill in this form)

- ○ not submitted yet - in early draft status
- ○ not submitted yet - in late draft status, just before submission
- ○ submitted to a journal but not reviewed yet
- ○ submitted to a journal and after receiving initial reviewer comments
- ◉ submitted to a journal and accepted, but not published yet
- ○ published
- ○ Other:

Journal *

If you already know where you will submit this paper (or if it is already submitted), please provide the journal name (if it is not JMIR, provide the journal name under "other")

- ○ not submitted yet / unclear where I will submit this
- ◉ Journal of Medical Internet Research (JMIR)
- ○ JMIR mHealth and UHealth
- ○ JMIR Serious Games
- ○ JMIR Mental Health
- ○ JMIR Public Health
- ○ JMIR Formative Research
- ○ Other JMIR sister journal
- ○ Other:

Is this a full powered effectiveness trial or a pilot/feasibility trial? *

- ( ) Pilot/feasibility
- (•) Fully powered

Manuscript tracking number *

If this is a JMIR submission, please provide the manuscript tracking number under "other" (The ms tracking number can be found in the submission acknowledgement email, or when you login as author in JMIR. If the paper is already published in JMIR, then the ms tracking number is the four-digit number at the end of the DOI, to be found at the bottom of each published article in JMIR)

- ( ) no ms number (yet) / not (yet) submitted to / published in JMIR
- (•) Other: JMIR ms#43740

TITLE AND ABSTRACT

1a) TITLE: Identification as a randomized trial in the title

1a) Does your paper address CONSORT item 1a? *

I.e does the title contain the phrase "Randomized Controlled Trial"? (if not, explain the reason under "other")

- (•) yes
- ( ) Other:

1a-i) Identify the mode of delivery in the title

Identify the mode of delivery. Preferably use “web-based” and/or “mobile” and/or “electronic game” in the title. Avoid ambiguous terms like “online”, “virtual”, “interactive”. Use “Internet-based” only if Intervention includes non-web-based Internet components (e.g. email), use “computer-based” or “electronic” only if offline products are used. Use “virtual” only in the context of “virtual reality” (3-D worlds). Use “online” only in the context of “online support groups”. Complement or substitute product names with broader terms for the class of products (such as “mobile” or “smart phone” instead of “iphone”), especially if the application runs on different platforms.

subitem not at all important

1 ○

2 ○

3 ○

4 ○

5 ◉

essential

Clear selection

Does your paper address subitem 1a-i? *

Copy and paste relevant sections from manuscript title (include quotes in quotation marks "like this" to indicate direct quotes from your manuscript), or elaborate on this item by providing additional information not in the ms, or briefly explain why the item is not applicable/relevant for your study

Yes, "A Web-based Sexual Assault, Alcohol Misuse and Bystander Intervention Program"

1a-ii) Non-web-based components or important co-interventions in title

Mention non-web-based components or important co-interventions in title, if any (e.g., “with telephone support”).

subitem not at all important

- 1 (selected)
- 2
- 3
- 4
- 5

essential

Clear selection

Does your paper address subitem 1a-ii?

Copy and paste relevant sections from manuscript title (include quotes in quotation marks "like this" to indicate direct quotes from your manuscript), or elaborate on this item by providing additional information not in the ms, or briefly explain why the item is not applicable/relevant for your study

The intervention does not involve any non-web-based components

1a-iii) Primary condition or target group in the title

Mention primary condition or target group in the title, if any (e.g., “for children with Type I Diabetes”) Example: A Web-based and Mobile Intervention with Telephone Support for Children with Type I Diabetes: Randomized Controlled Trial

subitem not at all important

- 1 ○
- 2 ○
- 3 ○
- 4 ○
- 5 ◉

essential

Clear selection

Does your paper address subitem 1a-iii? *

Copy and paste relevant sections from manuscript title (include quotes in quotation marks "like this" to indicate direct quotes from your manuscript), or elaborate on this item by providing additional information not in the ms, or briefly explain why the item is not applicable/relevant for your study

Yes, "A Web-based Sexual Violence, Alcohol Misuse, and Bystander Intervention Program for College Women"

1b) ABSTRACT: Structured summary of trial design, methods, results, and conclusions

NPT extension: Description of experimental treatment, comparator, care providers, centers, and blinding status.

1b-i) Key features/functionalities/components of the intervention and comparator in the METHODS section of the ABSTRACT

Mention key features/functionalities/components of the intervention and comparator in the abstract. If possible, also mention theories and principles used for designing the site. Keep in mind the needs of systematic reviewers and indexers by including important synonyms. (Note: Only report in the abstract what the main paper is reporting. If this information is missing from the main body of text, consider adding it)

subitem not at all important

1 ○

2 ○

3 ○

4 ○

5 ◉

essential

Clear selection

Does your paper address subitem 1b-i? *

Copy and paste relevant sections from the manuscript abstract (include quotes in quotation marks "like this" to indicate direct quotes from your manuscript), or elaborate on this item by providing additional information not in the ms, or briefly explain why the item is not applicable/relevant for your study

Yes, "Women aged 18 to 20 years were randomized to RealConsent (n=444) or to an attention-matched placebo control)(n=437)"

1b-ii) Level of human involvement in the METHODS section of the ABSTRACT

Clarify the level of human involvement in the abstract, e.g., use phrases like “fully automated” vs. “therapist/nurse/care provider/physician-assisted” (mention number and expertise of providers involved, if any). (Note: Only report in the abstract what the main paper is reporting. If this information is missing from the main body of text, consider adding it)

subitem not at all important

1 ○

2 ○

3 ○

4 ○

5 ◉

essential

Clear selection

Does your paper address subitem 1b-ii?

Copy and paste relevant sections from the manuscript abstract (include quotes in quotation marks "like this" to indicate direct quotes from your manuscript), or elaborate on this item by providing additional information not in the ms, or briefly explain why the item is not applicable/relevant for your study

"RealConsent is fully automated"

1b-iii) Open vs. closed, web-based (self-assessment) vs. face-to-face assessments in the METHODS section of the ABSTRACT

Mention how participants were recruited (online vs. offline), e.g., from an open access website or from a clinic or a closed online user group (closed usergroup trial), and clarify if this was a purely web-based trial, or there were face-to-face components (as part of the intervention or for assessment). Clearly say if outcomes were self-assessed through questionnaires (as common in web-based trials). Note: In traditional offline trials, an open trial (open-label trial) is a type of clinical trial in which both the researchers and participants know which treatment is being administered. To avoid confusion, use “blinded” or “unblinded” to indicated the level of blinding instead of “open”, as “open” in web-based trials usually refers to “open access” (i.e. participants can self-enrol). (Note: Only report in the abstract what the main paper is reporting. If this information is missing from the main body of text, consider adding it)

subitem not at all important

- 1 (selected)
- 2
- 3
- 4
- 5

essential

Clear selection

Does your paper address subitem 1b-iii?

Copy and paste relevant sections from the manuscript abstract (include quotes in quotation marks "like this" to indicate direct quotes from your manuscript), or elaborate on this item by providing additional information not in the ms, or briefly explain why the item is not applicable/relevant for your study

Additional information is provided in the manuscript that describes the online recruitment methods and that this trial was purely web-based with no face-to-face contact.

1b-iv) RESULTS section in abstract must contain use data

Report number of participants enrolled/assessed in each group, the use/uptake of the intervention (e.g., attrition/adherence metrics, use over time, number of logins etc.), in addition to primary/secondary outcomes. (Note: Only report in the abstract what the main paper is reporting. If this information is missing from the main body of text, consider adding it)

subitem not at all important

1 ○

2 ○

3 ○

4 ●

5 ○

essential

Clear selection

Does your paper address subitem 1b-iv?

Copy and paste relevant sections from the manuscript abstract (include quotes in quotation marks "like this" to indicate direct quotes from your manuscript), or elaborate on this item by providing additional information not in the ms, or briefly explain why the item is not applicable/relevant for your study

Yes, we report in the abstract the number of women assigned to each group, however, we do not report attrition in the abstract. Attrition by group is reported in the manuscript as per CONSORT.

1b-v) CONCLUSIONS/DISCUSSION in abstract for negative trials

Conclusions/Discussions in abstract for negative trials: Discuss the primary outcome - if the trial is negative (primary outcome not changed), and the intervention was not used, discuss whether negative results are attributable to lack of uptake and discuss reasons. (Note: Only report in the abstract what the main paper is reporting. If this information is missing from the main body of text, consider adding it)

subitem not at all important

- 1 (selected)
- 2
- 3
- 4
- 5

essential

Clear selection

Does your paper address subitem 1b-v?

Copy and paste relevant sections from the manuscript abstract (include quotes in quotation marks "like this" to indicate direct quotes from your manuscript), or elaborate on this item by providing additional information not in the ms, or briefly explain why the item is not applicable/relevant for your study

Yes, significant changes in the primary outcome were observed and reported in the Results section; thus, this was not a negative trial.

INTRODUCTION

2a) In INTRODUCTION: Scientific background and explanation of rationale

2a-i) Problem and the type of system/solution

Describe the problem and the type of system/solution that is object of the study: intended as stand-alone intervention vs. incorporated in broader health care program? Intended for a particular patient population? Goals of the intervention, e.g., being more cost-effective to other interventions, replace or complement other solutions? (Note: Details about the intervention are provided in "Methods" under 5)

subitem not at all important

1 ○

2 ○

3 ○

4 ○

5 ◉

essential

Clear selection

Does your paper address subitem 2a-i? *

Copy and paste relevant sections from the manuscript (include quotes in quotation marks "like this" to indicate direct quotes from your manuscript), or elaborate on this item by providing additional information not in the ms, or briefly explain why the item is not applicable/relevant for your study

Yes, "Sexual violence (SV) is a widespread, complex social and behavioral problem for which we currently have few comprehensive approaches to prevention, and even fewer for college women.[1-4] SV encompasses a range of behaviors on a continuum from "minor" behaviors (e.g., catcalling, verbal suggestions of intent to force someone to have sex) to more extreme behaviors (e.g., attempted or completed rape)...Despite decades of research, we have not moved the needle on campus SV"..."Furthermore, most prevention programs for both men and women are delivered through in-person small group settings, which limits the scope of dissemination...RealConsent (women's version), was developed using web-based and mobile technology and is an educational program aimed specifically at helping college-aged women develop protective behaviors against sexual violence."

2a-ii) Scientific background, rationale: What is known about the (type of) system

Scientific background, rationale: What is known about the (type of) system that is the object of the study (be sure to discuss the use of similar systems for other conditions/diagnoses, if appropiate), motivation for the study, i.e. what are the reasons for and what is the context for this specific study, from which stakeholder viewpoint is the study performed, potential impact of findings [2]. Briefly justify the choice of the comparator.

subitem not at all important

1 ○

2 ○

3 ○

4 ○

5 ◉

essential

Clear selection

Does your paper address subitem 2a-ii? *

Copy and paste relevant sections from the manuscript (include quotes in quotation marks "like this" to indicate direct quotes from your manuscript), or elaborate on this item by providing additional information not in the ms, or briefly explain why the item is not applicable/relevant for your study

Yes, "Although numerous existing prevention programs focus on reducing perpetration rates among men or improving bystander behaviors, there is a need for programs that combine multiple, comprehensive prevention strategies. [19-22] Additionally, one area neglected in the current intervention landscape are programs targeted specifically at educating and empowering women. While the majority of programs do and should focus on identifying and preventing risk for perpetration by men, there are actionable tools and skillsets that can equip women to advocate for themselves when entering the college landscape. [20,23-25] Protective drinking behavior is a teachable skillset that has been identified as a protective factor for sexual violence victimization. [26-31] Programs directed at women should also seek to reduce risk by increasing individual ability to perceive risk for victimization, identify dangerous dating situations, enhance self-defense skills, and improve assertive communication skills. Although some programs do currently exist that address alcohol as a risk factor for sexual violence and protective drinking behaviors, [23,27,32] there are no programs specifically designed for college women that include alcohol use as a central program component. Furthermore, most prevention programs for both men and women are delivered through in-person small group settings, which limits the scope of dissemination. RealConsent (women's version), was developed using web-based and mobile technology and is an educational program aimed specifically at helping college-aged women develop protective behaviors against sexual violence."

2b) In INTRODUCTION: Specific objectives or hypotheses

Does your paper address CONSORT subitem 2b? *

Copy and paste relevant sections from the manuscript (include quotes in quotation marks "like this" to indicate direct quotes from your manuscript), or elaborate on this item by providing additional information not in the ms, or briefly explain why the item is not applicable/relevant for your study

Yes, "In this study, RealConsent was evaluated for efficacy in reducing SV incidence, in affecting alcohol and dating risk and protective factors, in reducing alcohol misuse and in increasing bystander behavior among college women."

METHODS

3a) Description of trial design (such as parallel, factorial) including allocation ratio

Does your paper address CONSORT subitem 3a? *

Copy and paste relevant sections from the manuscript (include quotes in quotation marks "like this" to indicate direct quotes from your manuscript), or elaborate on this item by providing additional information not in the ms, or briefly explain why the item is not applicable/relevant for your study

Yes, A randomized controlled trial (RCT) (ClinicalTrials.gov: NCT03726437) was implemented at three universities in the Southeastern United States. Study procedures were approved by the primary investigator's university institutional review board...Potential participants were blinded to study hypotheses and told that the purpose of the study was to "examine the effectiveness of a 3-hour web-based program for incoming female freshmen." Once they provided informed consent, participants were asked to complete an online registration form, where they provided their contact information including their full name, email, address, and phone number. Then, participants were redirected to the baseline survey assessment. Participants received $30 for completing the baseline survey. Once participants completed the baseline survey, they were directed to an online survey that collected data on their email and institution, which was then used to randomize participants to one of the two study conditions. Stratified block randomization was implemented via Redcap to randomly assign participants to either RealConsent® or to an attention-matched placebo condition called Stress and Mood Management (Isagroup.com)."

3b) Important changes to methods after trial commencement (such as eligibility criteria), with reasons

Does your paper address CONSORT subitem 3b? *

Copy and paste relevant sections from the manuscript (include quotes in quotation marks "like this" to indicate direct quotes from your manuscript), or elaborate on this item by providing additional information not in the ms, or briefly explain why the item is not applicable/relevant for your study

No changes were made after trial commencement.

3b-i) Bug fixes, Downtimes, Content Changes

Bug fixes, Downtimes, Content Changes: ehealth systems are often dynamic systems. A description of changes to methods therefore also includes important changes made on the intervention or comparator during the trial (e.g., major bug fixes or changes in the functionality or content) (5-iii) and other “unexpected events” that may have influenced study design such as staff changes, system failures/downtimes, etc. [2].

subitem not at all important

- 1 (selected)
- 2
- 3
- 4
- 5

essential

Clear selection

Does your paper address subitem 3b-i?

Copy and paste relevant sections from the manuscript (include quotes in quotation marks "like this" to indicate direct quotes from your manuscript), or elaborate on this item by providing additional information not in the ms, or briefly explain why the item is not applicable/relevant for your study

No changes were made to the intervention or to the comparator during the trial.

4a) Eligibility criteria for participants

Does your paper address CONSORT subitem 4a? *

Copy and paste relevant sections from the manuscript (include quotes in quotation marks "like this" to indicate direct quotes from your manuscript), or elaborate on this item by providing additional information not in the ms, or briefly explain why the item is not applicable/relevant for your study

Yes, "Eligible participants were students identifying as women, 18-20 years of age, single, and entering their first year of college."

4a-i) Computer / Internet literacy

Computer / Internet literacy is often an implicit “de facto” eligibility criterion - this should be explicitly clarified.

subitem not at all important

- 1 (selected)
- 2
- 3
- 4
- 5

essential

Clear selection

Does your paper address subitem 4a-i?

Copy and paste relevant sections from the manuscript (include quotes in quotation marks "like this" to indicate direct quotes from your manuscript), or elaborate on this item by providing additional information not in the ms, or briefly explain why the item is not applicable/relevant for your study

As participants were first-year college women, recruited from three universities, via email, computer literacy was not an issue.

4a-ii) Open vs. closed, web-based vs. face-to-face assessments:

Open vs. closed, web-based vs. face-to-face assessments: Mention how participants were recruited (online vs. offline), e.g., from an open access website or from a clinic, and clarify if this was a purely web-based trial, or there were face-to-face components (as part of the intervention or for assessment), i.e., to what degree got the study team to know the participant. In online-only trials, clarify if participants were quasi-anonymous and whether having multiple identities was possible or whether technical or logistical measures (e.g., cookies, email confirmation, phone calls) were used to detect/prevent these.

subitem not at all important

1 ○

2 ○

3 ○

4 ○

5 ◉

essential

Clear selection

Does your paper address subitem 4a-ii? *

Copy and paste relevant sections from the manuscript (include quotes in quotation marks "like this" to indicate direct quotes from your manuscript), or elaborate on this item by providing additional information not in the ms, or briefly explain why the item is not applicable/relevant for your study

Yes, "An online sample N=881 first-year women college students were recruited using email contact lists provided by each university's Registrar's office. An email was sent to potential participants containing a description of the study with a link to an online survey delivered via Qualtrics to complete an eligibility screener. If individuals met eligibility criteria, they were then redirected to another online survey to complete the informed consent form and electronically provide their consent to participate. Potential participants were blinded to study hypotheses and told that the purpose of the study was to "examine the effectiveness of a 3-hour web-based program for incoming first-year women students."

4a-iii) Information giving during recruitment

Information given during recruitment. Specify how participants were briefed for recruitment and in the informed consent procedures (e.g., publish the informed consent documentation as appendix, see also item X26), as this information may have an effect on user self-selection, user expectation and may also bias results.

subitem not at all important

1 ○

2 ○

3 ○

4 ◉

5 ○

essential

Clear selection

Does your paper address subitem 4a-iii?

Copy and paste relevant sections from the manuscript (include quotes in quotation marks "like this" to indicate direct quotes from your manuscript), or elaborate on this item by providing additional information not in the ms, or briefly explain why the item is not applicable/relevant for your study

Yes, "Potential participants were blinded to study hypotheses and told that the purpose of the study was to “examine the effectiveness of a 3-hour web-based program for incoming first-year women students .”

4b) Settings and locations where the data were collected

Does your paper address CONSORT subitem 4b? *

Copy and paste relevant sections from the manuscript (include quotes in quotation marks "like this" to indicate direct quotes from your manuscript), or elaborate on this item by providing additional information not in the ms, or briefly explain why the item is not applicable/relevant for your study

Yes, "A randomized controlled trial (RCT) (ClinicalTrials.gov: NCT03726437) was implemented at three universities in the Southeastern United States. Study procedures were approved by the primary investigator’s university institutional review board. Eligible participants were 18-20 years of age, identified as a woman, single, and entering their first year of college."

4b-i) Report if outcomes were (self-)assessed through online questionnaires

Clearly report if outcomes were (self-)assessed through online questionnaires (as common in web-based trials) or otherwise.

subitem not at all important

1 ○

2 ○

3 ○

4 ○

5 ◉

essential

Clear selection

Does your paper address subitem 4b-i? *

Copy and paste relevant sections from the manuscript (include quotes in quotation marks "like this" to indicate direct quotes from your manuscript), or elaborate on this item by providing additional information not in the ms, or briefly explain why the item is not applicable/relevant for your study

Yes, "Once they provided informed consent, participants were asked to complete an online registration form, where they provided their contact information including their full name, email, address, and phone number. Then, participants were redirected to the baseline survey assessment. Participants received $30 for completing the baseline survey. Once participants completed the baseline survey, they were directed to an online survey that collected data on their email and institution, which was then used to randomize participants to one of the two study conditions. "

4b-ii) Report how institutional affiliations are displayed

Report how institutional affiliations are displayed to potential participants [on ehealth media], as affiliations with prestigious hospitals or universities may affect volunteer rates, use, and reactions with regards to an intervention.(Not a required item – describe only if this may bias results)

subitem not at all important

1 (selected)

2

3

4

5

essential

Clear selection

Does your paper address subitem 4b-ii?

Copy and paste relevant sections from the manuscript (include quotes in quotation marks "like this" to indicate direct quotes from your manuscript), or elaborate on this item by providing additional information not in the ms, or briefly explain why the item is not applicable/relevant for your study

Your answer

5) The interventions for each group with sufficient details to allow replication, including how and when they were actually administered

5-i) Mention names, credential, affiliations of the developers, sponsors, and owners

Mention names, credential, affiliations of the developers, sponsors, and owners [6] (if authors/evaluators are owners or developer of the software, this needs to be declared in a “Conflict of interest” section or mentioned elsewhere in the manuscript).

subitem not at all important

1 ○

2 ○

3 ○

4 ○

5 ◉

essential

Clear selection

Does your paper address subitem 5-i?

Copy and paste relevant sections from the manuscript (include quotes in quotation marks "like this" to indicate direct quotes from your manuscript), or elaborate on this item by providing additional information not in the ms, or briefly explain why the item is not applicable/relevant for your study

Yes, "Acknowledgements
Research reported in this publication was supported by the National Institute on Alcohol Abuse and Alcoholism of the National Institutes of Health under award number [R42AA025817]. The authors also wish to acknowledge the significant contributions of Phylicia Stewart, Inertia Films and George Cavagnaro to intervention development and production.
Conflicts of Interest
LFS and AMS-M are developers of RealConsent but neither derived financial income from the web-based program."

5-ii) Describe the history/development process

Describe the history/development process of the application and previous formative evaluations (e.g., focus groups, usability testing), as these will have an impact on adoption/use rates and help with interpreting results.

subitem not at all important

1 ○

2 ○

3 ○

4 ○

5 ◉

essential

Clear selection

Does your paper address subitem 5-ii?

Copy and paste relevant sections from the manuscript (include quotes in quotation marks "like this" to indicate direct quotes from your manuscript), or elaborate on this item by providing additional information not in the ms, or briefly explain why the item is not applicable/relevant for your study

Yes, "In developing RealConsent, extensive formative research with the targeted population was conducted to assess the different contexts in which SV victimization occurs, how men and women express sexual interest and consent, reasons for alcohol use, protective strategies to avoid SV, victim-blaming, barriers to bystander intervention, and stereotypical gender roles. The results were used to inform the content, messaging, language and story lines for each segment and particularly for the serial drama episodes titled “Squad.” Once the intervention module content was developed on paper, prior to production, an additional round of focus groups was conducted to assess the acceptability and relevancy of the materials and scripts in terms of literacy, language, realism, presentation and delivery."

5-iii) Revisions and updating

Revisions and updating. Clearly mention the date and/or version number of the application/intervention (and comparator, if applicable) evaluated, or describe whether the intervention underwent major changes during the evaluation process, or whether the development and/or content was “frozen” during the trial. Describe dynamic components such as news feeds or changing content which may have an impact on the replicability of the intervention (for unexpected events see item 3b).

subitem not at all important

- 1 (selected)
- 2
- 3
- 4
- 5

essential

Clear selection

Does your paper address subitem 5-iii?

Copy and paste relevant sections from the manuscript (include quotes in quotation marks "like this" to indicate direct quotes from your manuscript), or elaborate on this item by providing additional information not in the ms, or briefly explain why the item is not applicable/relevant for your study

Your answer

5-iv) Quality assurance methods

Provide information on quality assurance methods to ensure accuracy and quality of information provided [1], if applicable.

subitem not at all important

1 (selected)

2

3

4

5

essential

Clear selection

Does your paper address subitem 5-iv?

Copy and paste relevant sections from the manuscript (include quotes in quotation marks "like this" to indicate direct quotes from your manuscript), or elaborate on this item by providing additional information not in the ms, or briefly explain why the item is not applicable/relevant for your study

Your answer

5-v) Ensure replicability by publishing the source code, and/or providing screenshots/screen-capture video, and/or providing flowcharts of the algorithms used

Ensure replicability by publishing the source code, and/or providing screenshots/screen-capture video, and/or providing flowcharts of the algorithms used. Replicability (i.e., other researchers should in principle be able to replicate the study) is a hallmark of scientific reporting.

subitem not at all important

1 (selected)

2

3

4

5

essential

Clear selection

Does your paper address subitem 5-v?

Copy and paste relevant sections from the manuscript (include quotes in quotation marks "like this" to indicate direct quotes from your manuscript), or elaborate on this item by providing additional information not in the ms, or briefly explain why the item is not applicable/relevant for your study

Your answer

5-vi) Digital preservation

Digital preservation: Provide the URL of the application, but as the intervention is likely to change or disappear over the course of the years; also make sure the intervention is archived (Internet Archive, webcitation.org, and/or publishing the source code or screenshots/videos alongside the article). As pages behind login screens cannot be archived, consider creating demo pages which are accessible without login.

subitem not at all important

1 (selected)

2

3

4

5

essential

Clear selection

Does your paper address subitem 5-vi?

Copy and paste relevant sections from the manuscript (include quotes in quotation marks "like this" to indicate direct quotes from your manuscript), or elaborate on this item by providing additional information not in the ms, or briefly explain why the item is not applicable/relevant for your study

Your answer

5-vii) Access

Access: Describe how participants accessed the application, in what setting/context, if they had to pay (or were paid) or not, whether they had to be a member of specific group. If known, describe how participants obtained “access to the platform and Internet” [1]. To ensure access for editors/reviewers/readers, consider to provide a “backdoor” login account or demo mode for reviewers/readers to explore the application (also important for archiving purposes, see vi).

subitem not at all important

1 ○

2 ○

3 ○

4 ○

5 ◉

essential

Clear selection

Does your paper address subitem 5-vii? *

Copy and paste relevant sections from the manuscript (include quotes in quotation marks "like this" to indicate direct quotes from your manuscript), or elaborate on this item by providing additional information not in the ms, or briefly explain why the item is not applicable/relevant for your study

Yes, "RealConsent was delivered via a password-protected Web portal that allowed women to access the program either via the web or their mobile phone."

5-viii) Mode of delivery, features/functionalities/components of the intervention and comparator, and the theoretical framework

Describe mode of delivery, features/functionalities/components of the intervention and comparator, and the theoretical framework [6] used to design them (instructional strategy [1], behaviour change techniques, persuasive features, etc., see e.g., [7, 8] for terminology). This includes an in-depth description of the content (including where it is coming from and who developed it) [1]," whether [and how] it is tailored to individual circumstances and allows users to track their progress and receive feedback" [6]. This also includes a description of communication delivery channels and – if computer-mediated communication is a component – whether communication was synchronous or asynchronous [6]. It also includes information on presentation strategies [1], including page design principles, average amount of text on pages, presence of hyperlinks to other resources, etc. [1].

subitem not at all important

1 ○

2 ○

3 ○

4 ○

5 ◉

essential

Clear selection

Does your paper address subitem 5-viii? *

Copy and paste relevant sections from the manuscript (include quotes in quotation marks "like this" to indicate direct quotes from your manuscript), or elaborate on this item by providing additional information not in the ms, or briefly explain why the item is not applicable/relevant for your study

Yes, "RealConsent was delivered via a password-protected Web portal that allowed women to access the program either via the web or their mobile phones. The program contains four 45-minute program modules for a total duration of 3 hours. Each module includes interactivity, didactic activities, and entertainment-education media. [39,40] Entertainment-education is an effective health communication strategy that combines or embeds educational messages into entertainment programs to bring about social and behavior change. [40] RealConsent® contains eight mini-episodes of a serial drama titled, “Squad” as its entertainment-education. To ensure relevancy and quality of “Squad,”, we worked with a professional scriptwriter to ensure that our dialogue was realistic and entertaining. In addition, we contracted with a professional film and video company, who had won several Emmy awards for their documentary films, to film, direct and edit our video segments. Consequently, the “Squad” serial drama garnered four Telly Awards. Telly awards honor excellence in video and television across all platforms. The Squad episodes allow for the modeling of positive behaviors and for illustrating both positive and negative outcome expectations related to alcohol misuse and bystander intervention. RealConsent includes ethnically and racially diverse actors in its filmed segments and in images accompanying didactic segments, and also includes representation of same-sex relationships. RealConsent® was programmed so that participants could not skip or click-through segments within each module without viewing the entire segment. In addition, the program contained an administrative component that allowed study staff to track participants' completion of the program.

Attention-Placebo Control Intervention

Stress and Mood Management is a web-based, multi-media health promotion program designed to help manage stress levels, prevent mood problems, and seek early identification and treatment for depression and anxiety developed by ISA Group. Each of four program modules is ≈30 minutes involving videos and interactive and didactic activities. Thus, it approximates RealConsent® in format and duration."

5-ix) Describe use parameters

Describe use parameters (e.g., intended “doses” and optimal timing for use). Clarify what instructions or recommendations were given to the user, e.g., regarding timing, frequency, heaviness of use, if any, or was the intervention used ad libitum.

subitem not at all important

- 1 ○
- 2 ○
- 3 ○
- 4 ◉
- 5 ○

essential

Clear selection

Does your paper address subitem 5-ix?

Copy and paste relevant sections from the manuscript (include quotes in quotation marks "like this" to indicate direct quotes from your manuscript), or elaborate on this item by providing additional information not in the ms, or briefly explain why the item is not applicable/relevant for your study

Yes, "Participants were encouraged to complete their assigned program within a week by offering a $10 incentive for completing a brief acceptability survey following each program module."

5-x) Clarify the level of human involvement

Clarify the level of human involvement (care providers or health professionals, also technical assistance) in the e-intervention or as co-intervention (detail number and expertise of professionals involved, if any, as well as “type of assistance offered, the timing and frequency of the support, how it is initiated, and the medium by which the assistance is delivered”. It may be necessary to distinguish between the level of human involvement required for the trial, and the level of human involvement required for a routine application outside of a RCT setting (discuss under item 21 – generalizability).

subitem not at all important

- 1 (selected)
- 2
- 3
- 4
- 5

essential

Clear selection

Does your paper address subitem 5-x?

Copy and paste relevant sections from the manuscript (include quotes in quotation marks "like this" to indicate direct quotes from your manuscript), or elaborate on this item by providing additional information not in the ms, or briefly explain why the item is not applicable/relevant for your study

As the program was self-paced and accessed via the web using a computer or mobile phone, the only interaction that occurred with participants was the email with the link and instructions on how to access their assigned program. Also, if they had technical difficulties, interactions to solve the issue would occur via email.

5-xi) Report any prompts/reminders used

Report any prompts/reminders used: Clarify if there were prompts (letters, emails, phone calls, SMS) to use the application, what triggered them, frequency etc. It may be necessary to distinguish between the level of prompts/reminders required for the trial, and the level of prompts/reminders for a routine application outside of a RCT setting (discuss under item 21 – generalizability).

subitem not at all important

- 1 ○
- 2 ○
- 3 ○
- 4 ◉
- 5 ○

essential

Clear selection

Does your paper address subitem 5-xi? *

Copy and paste relevant sections from the manuscript (include quotes in quotation marks "like this" to indicate direct quotes from your manuscript), or elaborate on this item by providing additional information not in the ms, or briefly explain why the item is not applicable/relevant for your study

Progress through intervention modules was monitored. Email reminders were sent to participants to complete the program within a week. Also, following the completion of each module, we would send participants a link to a brief, survey for which they received $10 for completing.

5-xii) Describe any co-interventions (incl. training/support)

Describe any co-interventions (incl. training/support): Clearly state any interventions that are provided in addition to the targeted eHealth intervention, as ehealth intervention may not be designed as stand-alone intervention. This includes training sessions and support [1]. It may be necessary to distinguish between the level of training required for the trial, and the level of training for a routine application outside of a RCT setting (discuss under item 21 – generalizability.

subitem not at all important

- 1 (selected)
- 2
- 3
- 4
- 5

essential

Clear selection

Does your paper address subitem 5-xii? *

Copy and paste relevant sections from the manuscript (include quotes in quotation marks "like this" to indicate direct quotes from your manuscript), or elaborate on this item by providing additional information not in the ms, or briefly explain why the item is not applicable/relevant for your study

RealConsent is a stand-alone program that can be delivered to users without the need for additional training or support.

6a) Completely defined pre-specified primary and secondary outcome measures, including how and when they were assessed

Does your paper address CONSORT subitem 6a? *

Copy and paste relevant sections from the manuscript (include quotes in quotation marks "like this" to indicate direct quotes from your manuscript), or elaborate on this item by providing additional information not in the ms, or briefly explain why the item is not applicable/relevant for your study

Yes, "Primary Outcome Variable
Sexual Assault Victimization. Sexual assault victimization was assessed with Koss et al.'s Revised Sexual Experience Survey (SES). [41] Behaviorally specific language was used to describe the unwanted sexual experience outcomes and tactics. Types of unwanted sexual behavior assessed included sexual contact (e.g., fondling) and attempted or completed penetration (oral, vaginal, or anal). The tactics included two forms of verbal coercion including (1) telling lies, verbal threats, making promises known to be untrue, or using verbal pressure and (2) showing displeasure, criticizing, or getting angry; incapacitation (i.e., taking advantage when the participant was "too drunk or out of it" to stop what was happening); and two forms of physical force including (1) threatening physical force and (2) use of physical force. Participants were asked how often each sexual experience was obtained by each tactic with response options ranging from 0 (never) to 3 (three or more times). At baseline, "in the past 12 months" was used as the time reference and at 6-months follow-up, "since viewing the web-based program" was the time reference. Sexual victimization was analyzed in two ways as recommended by Davis et al [42] and which takes into consideration the severity of the assault (e.g., "fondling" vs. "completed vaginal rape") and also the tactic used (e.g., "alcohol incapacitated" vs. "physical force") in addition to the frequency. The combined outcomes separated tactics scale (COSTS) was measured as a continuous construct with a range of 0-63 where six severity ranks were used. For the rape outcomes that had the same tactic (e.g., "attempted rape by force" and "completed rape by force"), each was given the same severity rank. Each severity score was multiplied by the frequency and then summed. The separated outcomes separated tactics (SOTS) was measured as a continuous construct with a range of 0-135 where each outcome by each tactic was ranked by severity from 1 (sexual contact with verbal coercion) to 9 (completed rape by force) and then multiplied by the frequency. [42]

Secondary Outcomes
Alcohol Protective Behaviors. Alcohol protective behaviors were assessed with 15 items from the Protective Behavioral Strategies Survey,[43] with answer choices ranging on a 5-point scale (1) Always to (5) Never. Participants were asked while using alcohol or "partying" whether they engaged in alcohol-related protective behaviors (e.g., "determine not to exceed a set number of drinks," "avoid mixing different types of alcohol," and "know where your drink had been at all times"). For the current study, the scale showed adequate reliability (α =.86). Items were summed for a total score for engaging in protective behaviors.

Dating Risk Behaviors. Dating risk behaviors were assessed using the Dating Behavior Survey, which consists of 15 items assessing the situational variables including alcohol use that have been found to be related to acquaintance rape. [44] Participants indicated how often they engaged in situational behaviors that would put them at risk (e.g., On the first few dates..., "I consume alcohol or drugs," or " my partner and I do things that allow us to spend time alone together"). Answer choices ranged on a 5-point scale (1) Never to (5) Always. For the current study, reliability was adequate (α =.71). Responses were summed for a total score for risk-related dating behavior.

Alcohol Use. Alcohol use was assessed using several items from the Daily Drinking Questionnaire – Revised. [45] Participants were asked to report: the number of times they consumed four or more alcoholic drinks in one sitting in the last 30 days (binge drinking), the number of drinks plus the number of hours they drank each day for a typical week [illegible]

(average # of drinks per hour), and the number of drinks consumed for one occasion where they "drank the most" during the past 30 days (heavy drinking).

Bystander Behavior. Bystander behavior was assessed using the 20-item Bystander Behavior Scale [46]. Items assessed whether participants engaged in bystander behaviors in the past three months and included items such as, "If I saw someone taking a very intoxicated person up to their room, I said something and asked what the friend was doing." Response options included how many times they intervened (e.g., 1, 2, 3+) or "no opportunity." For the current study, reliability was adequate (α =.84). Responses were summed for a total score for bystander behavior."

6a-i) Online questionnaires: describe if they were validated for online use and apply CHERRIES items to describe how the questionnaires were designed/deployed

If outcomes were obtained through online questionnaires, describe if they were validated for online use and apply CHERRIES items to describe how the questionnaires were designed/deployed [9].

subitem not at all important

- 1 (selected)
- 2
- 3
- 4
- 5

essential

Clear selection

Does your paper address subitem 6a-i?

Copy and paste relevant sections from manuscript text

All questionnaires used have been validated and used in previous studies and were programmed using Qualtrics survey platform.

6a-ii) Describe whether and how “use” (including intensity of use/dosage) was defined/measured/monitored

Describe whether and how “use” (including intensity of use/dosage) was defined/measured/monitored (logins, logfile analysis, etc.). Use/adoption metrics are important process outcomes that should be reported in any ehealth trial.

subitem not at all important

1 ○

2 ○

3 ○

4 ○

5 ◉

essential

Clear selection

Does your paper address subitem 6a-ii?

Copy and paste relevant sections from manuscript text

Dosage was monitored and defined as the completion of each of the modules constituting each web-based program. 100% dosage was completion of all 4 modules; 75% dosage was completion of 3/4 modules, 50% dosage was completion of 2/4 modules, 25% dosage was completion of 1/4 modules, and 0% was 0/4 modules. Dosage was monitored through each of the intervention's web-based platform. Dosage is reported in the study flowchart.

6a-iii) Describe whether, how, and when qualitative feedback from participants was obtained

Describe whether, how, and when qualitative feedback from participants was obtained (e.g., through emails, feedback forms, interviews, focus groups).

subitem not at all important

1 ○

2 ○

3 ○

4 ◉

5 ○

essential

Clear selection

Does your paper address subitem 6a-iii?

Copy and paste relevant sections from manuscript text

Following completion of each intervention module, participants were sent a brief survey assessing their feedback.

6b) Any changes to trial outcomes after the trial commenced, with reasons

Does your paper address CONSORT subitem 6b? *

Copy and paste relevant sections from the manuscript (include quotes in quotation marks "like this" to indicate direct quotes from your manuscript), or elaborate on this item by providing additional information not in the ms, or briefly explain why the item is not applicable/relevant for your study

No changes were made to trial outcomes after trial commenced.

7a) How sample size was determined

NPT: When applicable, details of whether and how the clustering by care provides or centers was addressed

7a-i) Describe whether and how expected attrition was taken into account when calculating the sample size

Describe whether and how expected attrition was taken into account when calculating the sample size.

subitem not at all important

1 ○

2 ○

3 ○

4 ○

5 ◉

essential

Clear selection

Does your paper address subitem 7a-i?

Copy and paste relevant sections from manuscript title (include quotes in quotation marks "like this" to indicate direct quotes from your manuscript), or elaborate on this item by providing additional information not in the ms, or briefly explain why the item is not applicable/relevant for your study

Yes, "Sample size calculations for the primary outcome was estimated to guarantee that power would be at least .80 for the detection of a small to moderate effect size (Cohen's h ≥ .35). With two study groups, we estimated a needed sample size of at least 670; however, we factored in anticipated 20% attrition, which resulted in enrolling at least 750 participants (375 in each group) to increase power."

7b) When applicable, explanation of any interim analyses and stopping guidelines

Does your paper address CONSORT subitem 7b? *

Copy and paste relevant sections from the manuscript (include quotes in quotation marks "like this" to indicate direct quotes from your manuscript), or elaborate on this item by providing additional information not in the ms, or briefly explain why the item is not applicable/relevant for your study

No interim analyses were performed. As this was to evaluate a behavioral intervention, there were no stopping guidelines.

8a) Method used to generate the random allocation sequence
NPT: When applicable, how care providers were allocated to each trial group

Does your paper address CONSORT subitem 8a? *

Copy and paste relevant sections from the manuscript (include quotes in quotation marks "like this" to indicate direct quotes from your manuscript), or elaborate on this item by providing additional information not in the ms, or briefly explain why the item is not applicable/relevant for your study

SAS statistical software was used to generate the stratified, block randomization sequence to ensure equal numbers between treatment and control groups and proportionate numbers from each of the three universities.

8b) Type of randomisation; details of any restriction (such as blocking and block size)

Does your paper address CONSORT subitem 8b? *

Copy and paste relevant sections from the manuscript (include quotes in quotation marks "like this" to indicate direct quotes from your manuscript), or elaborate on this item by providing additional information not in the ms, or briefly explain why the item is not applicable/relevant for your study

Yes, "Stratified block randomization was implemented via Redcap to randomly assign participants to either RealConsent® or to an attention-matched placebo condition called Stress and Mood Management (Isagroup.com)."

9) Mechanism used to implement the random allocation sequence (such as sequentially numbered containers), describing any steps taken to conceal the sequence until interventions were assigned

Does your paper address CONSORT subitem 9? *

Copy and paste relevant sections from the manuscript (include quotes in quotation marks "like this" to indicate direct quotes from your manuscript), or elaborate on this item by providing additional information not in the ms, or briefly explain why the item is not applicable/relevant for your study

The stratified randomization sequence was uploaded to RedCap, which was used to randomize each participant by the study project director.

10) Who generated the random allocation sequence, who enrolled participants, and who assigned participants to interventions

Does your paper address CONSORT subitem 10? *

Copy and paste relevant sections from the manuscript (include quotes in quotation marks "like this" to indicate direct quotes from your manuscript), or elaborate on this item by providing additional information not in the ms, or briefly explain why the item is not applicable/relevant for your study

The study biostatistician generated the random allocation sequence. Participants enrolled in the study online. Once enrolled, participants completed the online baseline assessment, then the study project director used RedCap to access the sequence and randomize each participant.

11a) If done, who was blinded after assignment to interventions (for example, participants, care providers, those assessing outcomes) and how

NPT: Whether or not administering co-interventions were blinded to group assignment

11a-i) Specify who was blinded, and who wasn't

Specify who was blinded, and who wasn't. Usually, in web-based trials it is not possible to blind the participants [1, 3] (this should be clearly acknowledged), but it may be possible to blind outcome assessors, those doing data analysis or those administering co-interventions (if any).

subitem not at all important

1 ○

2 ○

3 ○

4 ○

5 ◉

essential

Clear selection

Does your paper address subitem 11a-i? *

Copy and paste relevant sections from the manuscript (include quotes in quotation marks "like this" to indicate direct quotes from your manuscript), or elaborate on this item by providing additional information not in the ms, or briefly explain why the item is not applicable/relevant for your study

Yes, "Potential participants were blinded to study hypotheses and told that the purpose of the study was to “examine the effectiveness of a 3-hour web-based program for incoming female freshmen.” In addition, they were informed that they would be randomized to view one of two different web-based programs. Each program covers topics related to college women's mental and physical health and safety. In addition, the online assessment included additional measures directly related to the comparator program such as stress level, anxiety, and mood. The biostatistician was also blinded in that study condition was assigned either "a" or "b" in all data files.

11a-ii) Discuss e.g., whether participants knew which intervention was the “intervention of interest” and which one was the “comparator”

Informed consent procedures (4a-ii) can create biases and certain expectations - discuss e.g., whether participants knew which intervention was the “intervention of interest” and which one was the “comparator”.

subitem not at all important

1 ○

2 ○

3 ○

4 ○

5 ◉

essential

Clear selection

Does your paper address subitem 11a-ii?

Copy and paste relevant sections from the manuscript (include quotes in quotation marks "like this" to indicate direct quotes from your manuscript), or elaborate on this item by providing additional information not in the ms, or briefly explain why the item is not applicable/relevant for your study

Yes, "Potential participants were blinded to study hypotheses and told that the purpose of the study was to “examine the effectiveness of a 3-hour web-based program for incoming female freshmen.” In addition, they were informed that they would be randomized to view one of two different web-based programs. Each program covers topics related to college women's mental and physical health and safety. In addition, the online assessment included additional measures directly related to the comparator program such as stress level, anxiety, and mood. As we did not interact directly with participants, we do not know for certain if they knew which program or if their assigned program was the program of interest.

11b) If relevant, description of the similarity of interventions

(this item is usually not relevant for ehealth trials as it refers to similarity of a placebo or sham intervention to a active medication/intervention)

Does your paper address CONSORT subitem 11b? *

Copy and paste relevant sections from the manuscript (include quotes in quotation marks "like this" to indicate direct quotes from your manuscript), or elaborate on this item by providing additional information not in the ms, or briefly explain why the item is not applicable/relevant for your study

Both interventions were web-based, involved some degree of interactivity, and were approximately 3 hours in duration.

12a) Statistical methods used to compare groups for primary and secondary outcomes

NPT: When applicable, details of whether and how the clustering by care providers or centers was addressed

Does your paper address CONSORT subitem 12a? *

Copy and paste relevant sections from the manuscript (include quotes in quotation marks "like this" to indicate direct quotes from your manuscript), or elaborate on this item by providing additional information not in the ms, or briefly explain why the item is not applicable/relevant for your study

Yes, "Analyses were performed on prespecified hypotheses for the primary outcome variable of sexual violence victimization using an intent-to-treat protocol in which participants were analyzed according to their assigned study conditions.[47] Additional analyses were also performed on several secondary outcomes including alcohol protective behaviors, dating risk behaviors and alcohol misuse using an intent-to-treat protocol. Statisticians were blinded to which group (“a” or “b”) was the experimental condition. An additional secondary outcome, bystander behavior, was also analyzed using a “dosage” protocol versus an intent-to-treat protocol. Participants who had completed 100% of the RealConsent program were compared to the control group participants plus those participants who had completed <100% of the program. Dosage was used to test for effects on bystander behavior as intervention content specific to bystander behavior was in the last module of the program.

Descriptive statistics were created for all study measures, with mean and standard deviation for continuous variables and frequency distribution for categorical variables. Comparisons were conducted of baseline findings across study characteristics and outcomes to determine if participants that completed the intervention were similar to those who did not. A substantial number of participants were expected to report not having an experience of sexual victimization, to not have engaged in bystander behavior, and/or not having previously consumed alcohol. To account for these zero occurrences, a comparison of the mean occurrence for each outcome across the baseline and 6-month follow-up time points was accomplished with a two-stage modeling process using zero-inflated regression models. In the first stage, a logistic regression model was used to model occurrence or not for each outcome, and in the second stage a Poisson or gamma regression model was used to model each outcome for those that had at least one occurrence. Sexual victimization and most of the alcohol consumption measurements were count outcomes and modeled with the Poisson distribution. The alcohol protective and dating risk behaviors scales were both continuous and reasonably symmetric. A multilevel model in the form of a general linear mixed model was used. The bystander behavior scale was skewed and assessed with logistic regression; heavy drinking outcomes were continuous and skewed and were modeled with a gamma distribution.

Participants were assessed at baseline and at 6-months follow-up. Repeated measurements on each participant results in within-subject correlation. This was accounted for by estimating each zero inflated model with population averaged effects using a marginal model and generalized estimating equations. Each zero-inflated marginal model included fixed effects to control for study site, race, ethnicity, place of living, relationship status, sexual orientation, engagement in athletics, job status, ever drank alcohol, time, study condition. A time by study condition interaction term was included in each model to assess and test for intervention effectiveness. The interaction term quantifies the relative change in the outcome over time across study conditions. Intervention effects were estimated with odds ratios for logistic models, incidence rate ratios for Poisson models, and regression coefficients for gamma models. The SAS Software system was used for all statistical analyses."

12a-i) Imputation techniques to deal with attrition / missing values

Imputation techniques to deal with attrition / missing values: Not all participants will use the intervention/comparator as intended and attrition is typically high in ehealth trials. Specify how participants who did not use the application or dropped out from the trial were treated in the statistical analysis (a complete case analysis is strongly discouraged, and simple imputation techniques such as LOCF may also be problematic [4]).

subitem not at all important

1 ○

2 ○

3 ○

4 ○

5 ◉

essential

Clear selection

Does your paper address subitem 12a-i? *

Copy and paste relevant sections from the manuscript (include quotes in quotation marks "like this" to indicate direct quotes from your manuscript), or elaborate on this item by providing additional information not in the ms, or briefly explain why the item is not applicable/relevant for your study

In this RCT, attrition was low (18%) and thus; imputation techniques were not implemented.

12b) Methods for additional analyses, such as subgroup analyses and adjusted analyses

Does your paper address CONSORT subitem 12b? *

Copy and paste relevant sections from the manuscript (include quotes in quotation marks "like this" to indicate direct quotes from your manuscript), or elaborate on this item by providing additional information not in the ms, or briefly explain why the item is not applicable/relevant for your study

Subgroup analyses were not performed. All models were adjusted for fixed effects: time, race/ethnicity, place of living, relationship status, sexual orientation, engagement in athletics, job status, ever drank alcohol, and ever on a date.

X26) REB/IRB Approval and Ethical Considerations [recommended as subheading under "Methods"] (not a CONSORT item)

X26-i) Comment on ethics committee approval

subitem not at all important

1 ○

2 ○

3 ○

4 ○

5 ◉

essential

Clear selection

Does your paper address subitem X26-i?

Copy and paste relevant sections from the manuscript (include quotes in quotation marks "like this" to indicate direct quotes from your manuscript), or elaborate on this item by providing additional information not in the ms, or briefly explain why the item is not applicable/relevant for your study

Yes, "This study was approved by the Georgia State University's Institutional Review Board (H19033)."

x26-ii) Outline informed consent procedures

Outline informed consent procedures e.g., if consent was obtained offline or online (how? Checkbox, etc.?), and what information was provided (see 4a-ii). See [6] for some items to be included in informed consent documents.

subitem not at all important

- 1 ○
- 2 ○
- 3 ○
- 4 ○
- 5 ◉

essential

Clear selection

Does your paper address subitem X26-ii?

Copy and paste relevant sections from the manuscript (include quotes in quotation marks "like this" to indicate direct quotes from your manuscript), or elaborate on this item by providing additional information not in the ms, or briefly explain why the item is not applicable/relevant for your study

Yes, "If individuals met eligibility criteria, they were then redirected to another online survey to complete the informed consent form and electronically provide their consent to participate."

The following was provided to potential participants during the informed consent procedure:

"The purpose of this study is to examine the effectiveness of a 3-hour web-based program for incoming female freshmen. If you decide to take part, you will take part in an online study. Participating in this study calls for about 5.5 hours of your time over the next six months. The study will involve:

- Completing a 45-minute online survey
- viewing a 4-part web-based program at your own pace (total time of program = 3 hours)
- Completing a 10-minute survey following each of the 4 parts
- Completing a second 45-minute online survey 6 months later

- The online survey will be on personal information (age, race, ethnicity, etc.), attitudes, health behaviors, dating behaviors, experiences of sexual violence, and perceptions of risk.
- The brief 10-minute surveys will ask your opinion on the program content.
- You will be randomized to view one of two different web-based programs. Each program covers topics related to female freshmen’s mental and physical health and safety.

☐ “Please check this box if you have read this section on the procedures of the research study.”

X26-iii) Safety and security procedures

Safety and security procedures, incl. privacy considerations, and any steps taken to reduce the likelihood or detection of harm (e.g., education and training, availability of a hotline)

subitem not at all important

1 ○

2 ○

3 ○

4 ○

5 ◉

essential

Clear selection

Does your paper address subitem X26-iii?

Copy and paste relevant sections from the manuscript (include quotes in quotation marks "like this" to indicate direct quotes from your manuscript), or elaborate on this item by providing additional information not in the ms, or briefly explain why the item is not applicable/relevant for your study

As part of the informed consent process, participants were told:
IV. Risks:
There is the possibility that taking part in this study may cause you to be nervous or stressed when answering some of the questions or viewing the program; you do not have to answer any questions you do not want to answer in the survey. Also, if you feel too anxious or stressed, you will be provided with the Principal Investigator’s phone number. If you need or want to talk to a trained therapist, you will be provided a toll-free 800-hotline number for you to call. You will also be given local university resources for you to contact.

RESULTS

13a) For each group, the numbers of participants who were randomly assigned, received intended treatment, and were analysed for the primary outcome

NPT: The number of care providers or centers performing the intervention in each group and the number of patients treated by each care provider in each center

Does your paper address CONSORT subitem 13a? *

Copy and paste relevant sections from the manuscript (include quotes in quotation marks "like this" to indicate direct quotes from your manuscript), or elaborate on this item by providing additional information not in the ms, or briefly explain why the item is not applicable/relevant for your study

Yes, "The recruitment process (See Figure 2) resulted in 4,473 first-year women college students who were screened for eligibility. Of those, 2,327 were not eligible, 349 did not undergo the informed consent process, 8 declined to participate, 908 did not enroll for other reasons. N=881 consented, completed baseline and were then randomized. At six-months, a total of 161 participants (18.3%) were lost to follow-up. Chi-square results indicated there was not differential attrition: 85 of 444 (18.9%) in the RealConsent condition versus 76 of 437 (17.4%) in the placebo comparison condition (P=.83).

13b) For each group, losses and exclusions after randomisation, together with reasons

Does your paper address CONSORT subitem 13b? (NOTE: Preferably, this is shown in a CONSORT flow diagram) *

Copy and paste relevant sections from the manuscript (include quotes in quotation marks "like this" to indicate direct quotes from your manuscript), or elaborate on this item by providing additional information not in the ms, or briefly explain why the item is not applicable/relevant for your study

Yes, "The recruitment process (See Figure 2) resulted in 4,473 first-year women college students who were screened for eligibility. Of those, 2,327 were not eligible, 349 did not undergo the informed consent process, 8 declined to participate, 908 did not enroll for other reasons. N=881 consented, completed baseline and were then randomized. At six-months, a total of 161 participants (18.3%) were lost to follow-up. Chi-square results indicated there was not differential attrition: 85 of 444 (18.9%) in the RealConsent condition versus 76 of 437 (17.4%) in the placebo comparison condition (P=.83)."

13b-i) Attrition diagram

Strongly recommended: An attrition diagram (e.g., proportion of participants still logging in or using the intervention/comparator in each group plotted over time, similar to a survival curve) or other figures or tables demonstrating usage/dose/engagement.

subitem not at all important

1 ○

2 ○

3 ○

4 ○

5 ◉

essential

Clear selection

Does your paper address subitem 13b-i?

Copy and paste relevant sections from the manuscript or cite the figure number if applicable (include quotes in quotation marks "like this" to indicate direct quotes from your manuscript), or elaborate on this item by providing additional information not in the ms, or briefly explain why the item is not applicable/relevant for your study

Figure 2 in the manuscript is the CONSORT study flowchart with attrition by group and also dosage of the intervention components by group shown.

14a) Dates defining the periods of recruitment and follow-up

Does your paper address CONSORT subitem 14a? *

Copy and paste relevant sections from the manuscript (include quotes in quotation marks "like this" to indicate direct quotes from your manuscript), or elaborate on this item by providing additional information not in the ms, or briefly explain why the item is not applicable/relevant for your study

Yes, "Active recruitment began in October 2018 and ended in February 2019." Participants were asked to complete a follow-up survey six months following the baseline survey.

14a-i) Indicate if critical "secular events" fell into the study period

Indicate if critical "secular events" fell into the study period, e.g., significant changes in Internet resources available or "changes in computer hardware or Internet delivery resources"

subitem not at all important

- 1 (selected)
- 2
- 3
- 4
- 5

essential

Clear selection

Does your paper address subitem 14a-i?

Copy and paste relevant sections from the manuscript (include quotes in quotation marks "like this" to indicate direct quotes from your manuscript), or elaborate on this item by providing additional information not in the ms, or briefly explain why the item is not applicable/relevant for your study

There were not any secular events affecting the trial during the study period.

14b) Why the trial ended or was stopped (early)

Does your paper address CONSORT subitem 14b? *

Copy and paste relevant sections from the manuscript (include quotes in quotation marks "like this" to indicate direct quotes from your manuscript), or elaborate on this item by providing additional information not in the ms, or briefly explain why the item is not applicable/relevant for your study

The trial ended following the completion of the 6-month follow-up and was not stopped early.

15) A table showing baseline demographic and clinical characteristics for each group

NPT: When applicable, a description of care providers (case volume, qualification, expertise, etc.) and centers (volume) in each group

Does your paper address CONSORT subitem 15? *

Copy and paste relevant sections from the manuscript (include quotes in quotation marks "like this" to indicate direct quotes from your manuscript), or elaborate on this item by providing additional information not in the ms, or briefly explain why the item is not applicable/relevant for your study

Yes "Table 1 provides data on the breakdown of sociodemographic variables and outcome variables by study condition."

15-i) Report demographics associated with digital divide issues

In ehealth trials it is particularly important to report demographics associated with digital divide issues, such as age, education, gender, social-economic status, computer/Internet/ehealth literacy of the participants, if known.

subitem not at all important

1 ○

2 ○

3 ○

4 ○

5 ◉

essential

Clear selection

Does your paper address subitem 15-i? *

Copy and paste relevant sections from the manuscript (include quotes in quotation marks "like this" to indicate direct quotes from your manuscript), or elaborate on this item by providing additional information not in the ms, or briefly explain why the item is not applicable/relevant for your study

Yes, for this RCT, only first-year women college students who matriculated at one of three chosen universities in the southeastern U.S. were eligible to participate. We report the following demographics for the sample:

"Being aged 18 to 20 years was an eligibility criterion; thus, the sample mirrored this age range. Most of the participants (642/881, 72.9%) were aged 18 years, followed by those aged 19 years (230/881, 26.1%) and 20 years (9/881, 1%). The racial breakdown of participants was as follows: American Indian or Alaska Native (6/881, 0.7%), Asian (176/881, 20%), Black or African American (213/881, 24.2%), Native Hawaiian or Pacific Islander (3/881, 0.3%), White (394/881, 44.7%), biracial or multiracial (76/881, 8.6%), and other (13/881, 1.5%). Hispanic or Latinx participants constituted 12.3% (108/881) of the sample. In terms of gender identity, most of the participants (873/881, 99.1%) identified as woman, and 0.9% (8/881) identified as nonconforming or nonbinary. Regarding sexual orientation, most of the participants (724/881, 82.2%) identified as heterosexual, 11.4% (100/881) identified as bisexual, 2.5% (22/881) identified as gay or lesbian, 1.5% (13/881) as queer, and 2.5% (22/881) as other"

16) For each group, number of participants (denominator) included in each analysis and whether the analysis was by original assigned groups

16-i) Report multiple “denominators” and provide definitions

Report multiple “denominators” and provide definitions: Report N’s (and effect sizes) “across a range of study participation [and use] thresholds” [1], e.g., N exposed, N consented, N used more than x times, N used more than y weeks, N participants “used” the intervention/comparator at specific pre-defined time points of interest (in absolute and relative numbers per group). Always clearly define “use” of the intervention.

subitem not at all important

1 ○

2 ○

3 ○

4 ○

5 ◉

essential

Clear selection

Does your paper address subitem 16-i? *

Copy and paste relevant sections from the manuscript (include quotes in quotation marks "like this" to indicate direct quotes from your manuscript), or elaborate on this item by providing additional information not in the ms, or briefly explain why the item is not applicable/relevant for your study

Yes, each of the N"s for each stage of the recruitment process, follow-up, and completion of intervention modules are presented in the CONSORT study flowchart.

16-ii) Primary analysis should be intent-to-treat

Primary analysis should be intent-to-treat, secondary analyses could include comparing only “users”, with the appropriate caveats that this is no longer a randomized sample (see 18-i).

subitem not at all important

1 ○

2 ○

3 ○

4 ○

5 ◉

essential

Clear selection

Does your paper address subitem 16-ii?

Copy and paste relevant sections from the manuscript (include quotes in quotation marks "like this" to indicate direct quotes from your manuscript), or elaborate on this item by providing additional information not in the ms, or briefly explain why the item is not applicable/relevant for your study

Yes, "Analyses were performed on prespecified hypotheses for the primary outcome variable of sexual violence victimization using an intent-to-treat protocol in which participants were analyzed according to their assigned study conditions.[47] Additional analyses were also performed on several secondary outcomes including alcohol protective behaviors, dating risk behaviors and alcohol misuse using an intent-to-treat protocol. Statisticians were blinded to which group (“a” or “b”) was the experimental condition. An additional secondary outcome, bystander behavior, was also analyzed using a “dosage” protocol versus an intent-to-treat protocol. Participants who had completed 100% of the RealConsent program were compared to the control group participants plus those participants who had completed <100% of the program. Dosage was used to test for effects on bystander behavior as intervention content specific to bystander behavior was in the last module of the program."

17a) For each primary and secondary outcome, results for each group, and the estimated effect size and its precision (such as 95% confidence interval)

Does your paper address CONSORT subitem 17a? *

Copy and paste relevant sections from the manuscript (include quotes in quotation marks "like this" to indicate direct quotes from your manuscript), or elaborate on this item by providing additional information not in the ms, or briefly explain why the item is not applicable/relevant for your study

Yes, for each primary and secondary outcome analyzed, results are presented for each group with corresponding estimated effect size, i.e., odds ratios for logistic models, incidence rate ratios for Poisson models, and regression coefficients for gamma models, and their corresponding 95% CI.

17a-i) Presentation of process outcomes such as metrics of use and intensity of use

In addition to primary/secondary (clinical) outcomes, the presentation of process outcomes such as metrics of use and intensity of use (dose, exposure) and their operational definitions is critical. This does not only refer to metrics of attrition (13-b) (often a binary variable), but also to more continuous exposure metrics such as "average session length". These must be accompanied by a technical description how a metric like a "session" is defined (e.g., timeout after idle time) [1] (report under item 6a).

subitem not at all important

1 ○

2 ○

3 ○

4 ○

5 ◉

essential

Clear selection

Does your paper address subitem 17a-i?

Copy and paste relevant sections from the manuscript (include quotes in quotation marks "like this" to indicate direct quotes from your manuscript), or elaborate on this item by providing additional information not in the ms, or briefly explain why the item is not applicable/relevant for your study

Yes, the paper reports dosage received for each intervention and attrition.

17b) For binary outcomes, presentation of both absolute and relative effect sizes is recommended

Does your paper address CONSORT subitem 17b? *

Copy and paste relevant sections from the manuscript (include quotes in quotation marks "like this" to indicate direct quotes from your manuscript), or elaborate on this item by providing additional information not in the ms, or briefly explain why the item is not applicable/relevant for your study

Yes, we present relative effect sizes for binary outcomes. The study's primary objective is to reduce risk of sexual violence victimization while the secondary outcomes are reducing hazardous drinking and increasing protective behaviors; thus, absolute effect sizes are not relevant as we are not examining the presence or absence of a disease outcome.

18) Results of any other analyses performed, including subgroup analyses and adjusted analyses, distinguishing pre-specified from exploratory

Does your paper address CONSORT subitem 18? *

Copy and paste relevant sections from the manuscript (include quotes in quotation marks "like this" to indicate direct quotes from your manuscript), or elaborate on this item by providing additional information not in the ms, or briefly explain why the item is not applicable/relevant for your study

Results are presented for pre-specified adjusted analyses. No other analyses were performed.

18-i) Subgroup analysis of comparing only users

A subgroup analysis of comparing only users is not uncommon in ehealth trials, but if done, it must be stressed that this is a self-selected sample and no longer an unbiased sample from a randomized trial (see 16-iii).

subitem not at all important

1 ○

2 ○

3 ◉

4 ○

5 ○

essential

Clear selection

Does your paper address subitem 18-i?

Copy and paste relevant sections from the manuscript (include quotes in quotation marks "like this" to indicate direct quotes from your manuscript), or elaborate on this item by providing additional information not in the ms, or briefly explain why the item is not applicable/relevant for your study

For one secondary outcome, bystander behavior, analyses were performed using dosage as the content for this outcome was in the last module of the intervention.

19) All important harms or unintended effects in each group

(for specific guidance see CONSORT for harms)

Does your paper address CONSORT subitem 19? *

Copy and paste relevant sections from the manuscript (include quotes in quotation marks "like this" to indicate direct quotes from your manuscript), or elaborate on this item by providing additional information not in the ms, or briefly explain why the item is not applicable/relevant for your study

No unintended effects or harms were reported during the course of this trial.

19-i) Include privacy breaches, technical problems

Include privacy breaches, technical problems. This does not only include physical “harm” to participants, but also incidents such as perceived or real privacy breaches [1], technical problems, and other unexpected/unintended incidents. “Unintended effects” also includes unintended positive effects [2].

subitem not at all important

1 ○

2 ○

3 ○

4 ◉

5 ○

essential

Clear selection

Does your paper address subitem 19-i?

Copy and paste relevant sections from the manuscript (include quotes in quotation marks "like this" to indicate direct quotes from your manuscript), or elaborate on this item by providing additional information not in the ms, or briefly explain why the item is not applicable/relevant for your study

No privacy breaches, or technical problems were reported during the course of this trial.

19-ii) Include qualitative feedback from participants or observations from staff/researchers

Include qualitative feedback from participants or observations from staff/researchers, if available, on strengths and shortcomings of the application, especially if they point to unintended/unexpected effects or uses. This includes (if available) reasons for why people did or did not use the application as intended by the developers.

subitem not at all important

1 ○

2 ○

3 ○

4 ◉

5 ○

essential

Clear selection

Does your paper address subitem 19-ii?

Copy and paste relevant sections from the manuscript (include quotes in quotation marks "like this" to indicate direct quotes from your manuscript), or elaborate on this item by providing additional information not in the ms, or briefly explain why the item is not applicable/relevant for your study

Qualitative data was collected to assess participants' acceptability of each intervention module, but results are not reported in this manuscript.

DISCUSSION

22) Interpretation consistent with results, balancing benefits and harms, and considering other relevant evidence

NPT: In addition, take into account the choice of the comparator, lack of or partial blinding, and unequal expertise of care providers or centers in each group

22-i) Restate study questions and summarize the answers suggested by the data, starting with primary outcomes and process outcomes (use)

Restate study questions and summarize the answers suggested by the data, starting with primary outcomes and process outcomes (use).

subitem not at all important

1 ○

2 ○

3 ○

4 ○

5 ◉

essential

Clear selection

Does your paper address subitem 22-i? *

Copy and paste relevant sections from the manuscript (include quotes in quotation marks "like this" to indicate direct quotes from your manuscript), or elaborate on this item by providing additional information not in the ms, or briefly explain why the item is not applicable/relevant for your study

Yes, "This study is the first comprehensive SV risk reduction program specific to women that incorporates entertainment-education media into web-based and mobile technologies, is theoretically and empirically informed, embodies proven behavior change techniques, includes alcohol education as a central component, and integrates bystander education as well as self-defense training—all factors associated with SV risk reduction. The results from this RCT demonstrate significant changes in both primary and secondary outcomes among a racially diverse sample of first-year women college students. Among participants who had experienced any SV, RealConsent participants reported less exposure to SV (primary outcome) than control group participants (P<.001). This result suggests that RealConsent is effective for participants who are most at risk. Although we did not find significant results for our zero-inflated logistic model, which compared no exposure to SV to any exposure to SV by condition, we can speculate possible reasons for this null finding.

22-ii) Highlight unanswered new questions, suggest future research

Highlight unanswered new questions, suggest future research.

subitem not at all important

- 1 ○
- 2 ○
- 3 ○
- 4 ○
- 5 ● (selected)

essential

Clear selection

Does your paper address subitem 22-ii?

Copy and paste relevant sections from the manuscript (include quotes in quotation marks "like this" to indicate direct quotes from your manuscript), or elaborate on this item by providing additional information not in the ms, or briefly explain why the item is not applicable/relevant for your study

The Discussion section highlights future research stemming from the results.

20) Trial limitations, addressing sources of potential bias, imprecision, and, if relevant, multiplicity of analyses

20-i) Typical limitations in ehealth trials

Typical limitations in ehealth trials: Participants in ehealth trials are rarely blinded. Ehealth trials often look at a multiplicity of outcomes, increasing risk for a Type I error. Discuss biases due to non-use of the intervention/usability issues, biases through informed consent procedures, unexpected events.

subitem not at all important

1 ○

2 ○

3 ○

4 ○

5 ◉

essential

Clear selection

Does your paper address subitem 20-i? *

Copy and paste relevant sections from the manuscript (include quotes in quotation marks "like this" to indicate direct quotes from your manuscript), or elaborate on this item by providing additional information not in the ms, or briefly explain why the item is not applicable/relevant for your study

Yes, "Our trial had several limitations. First, our trial was conducted with first-year college women recruited from three universities located in the southeastern United States. Future research should test RealConsent among college women matriculated at universities located in other geographical areas of the United States. Second, although not extreme, we experienced some loss to follow-up in terms of completion of the respective web-based programs and follow-up survey. It is unclear what the potential reasons were for this loss to follow-up; however, previous research has shown that attrition in web-based trials may be higher than in-person trials. [67,69,70] Yet our observed attrition rates (overall 18%) are lower (e.g.,40%) [71] or in-line with (e.g., 18%) [72] other in-person SV risk reduction trials with similar follow-up periods. Third, although the RCT design controls for many threats to internal validity, this trial was implemented in the field versus a lab where it was impossible to control for all external events. "

21) Generalisability (external validity, applicability) of the trial findings

NPT: External validity of the trial findings according to the intervention, comparators, patients, and care providers or centers involved in the trial

21-i) Generalizability to other populations

Generalizability to other populations: In particular, discuss generalizability to a general Internet population, outside of a RCT setting, and general patient population, including applicability of the study results for other organizations

subitem not at all important

1 ○

2 ○

3 ◉

4 ○

5 ○

essential

Clear selection

Does your paper address subitem 21-i?

Copy and paste relevant sections from the manuscript (include quotes in quotation marks "like this" to indicate direct quotes from your manuscript), or elaborate on this item by providing additional information not in the ms, or briefly explain why the item is not applicable/relevant for your study

Yes, "First, our trial was conducted with first-year college women recruited from three universities located in the southeastern United States. Future research should test RealConsent® among college women matriculated at universities located in other geographical areas of the United States."

21-ii) Discuss if there were elements in the RCT that would be different in a routine application setting

Discuss if there were elements in the RCT that would be different in a routine application setting (e.g., prompts/reminders, more human involvement, training sessions or other co-interventions) and what impact the omission of these elements could have on use, adoption, or outcomes if the intervention is applied outside of a RCT setting.

subitem not at all important

1 ○

2 ◉

3 ○

4 ○

5 ○

essential

Clear selection

Does your paper address subitem 21-ii?

Copy and paste relevant sections from the manuscript (include quotes in quotation marks "like this" to indicate direct quotes from your manuscript), or elaborate on this item by providing additional information not in the ms, or briefly explain why the item is not applicable/relevant for your study

Your answer

OTHER INFORMATION

23) Registration number and name of trial registry

Does your paper address CONSORT subitem 23? *

Copy and paste relevant sections from the manuscript (include quotes in quotation marks "like this" to indicate direct quotes from your manuscript), or elaborate on this item by providing additional information not in the ms, or briefly explain why the item is not applicable/relevant for your study

"ClinicalTrials.gov Identifier: NCT03726437"

24) Where the full trial protocol can be accessed, if available

Does your paper address CONSORT subitem 24? *

Cite a Multimedia Appendix, other reference, or copy and paste relevant sections from the manuscript (include quotes in quotation marks "like this" to indicate direct quotes from your manuscript), or elaborate on this item by providing additional information not in the ms, or briefly explain why the item is not applicable/relevant for your study

The trial protocol can be accessed through ClinicalTrials.gov Identifier: NCT03726437

25) Sources of funding and other support (such as supply of drugs), role of funders

Does your paper address CONSORT subitem 25? *

Copy and paste relevant sections from the manuscript (include quotes in quotation marks "like this" to indicate direct quotes from your manuscript), or elaborate on this item by providing additional information not in the ms, or briefly explain why the item is not applicable/relevant for your study

Yes, "Research reported in this publication was supported by the National Institute on Alcohol Abuse and Alcoholism of the National Institutes of Health (R42AA025817)."

X27) Conflicts of Interest (not a CONSORT item)

X27-i) State the relation of the study team towards the system being evaluated

In addition to the usual declaration of interests (financial or otherwise), also state the relation of the study team towards the system being evaluated, i.e., state if the authors/evaluators are distinct from or identical with the developers/sponsors of the intervention.

subitem not at all important

1 ○

2 ○

3 ○

4 ○

5 ◉

essential

Clear selection

Does your paper address subitem X27-i?

Copy and paste relevant sections from the manuscript (include quotes in quotation marks "like this" to indicate direct quotes from your manuscript), or elaborate on this item by providing additional information not in the ms, or briefly explain why the item is not applicable/relevant for your study

Yes, "LFS and AMS-M are the developers of RealConsent, but neither derived financial income from the web-based program."

About the CONSORT EHEALTH checklist

As a result of using this checklist, did you make changes in your manuscript? *

- ( ) yes, major changes
- (x) yes, minor changes
- ( ) no

What were the most important changes you made as a result of using this checklist?

I made minor changes to the abstract.

How much time did you spend on going through the checklist INCLUDING making changes in your manuscript *

I spend approximately 4 hours completing this checklist.

As a result of using this checklist, do you think your manuscript has improved? *

- (x) yes
- ( ) no
- ( ) Other:

Would you like to become involved in the CONSORT EHEALTH group?

This would involve for example becoming involved in participating in a workshop and writing an "Explanation and Elaboration" document

- ( ) yes
- (•) no
- ( ) Other:

Clear selection

Any other comments or questions on CONSORT EHEALTH

Your answer

STOP - Save this form as PDF before you click submit

To generate a record that you filled in this form, we recommend to generate a PDF of this page (on a Mac, simply select "print" and then select "print as PDF") before you submit it.

When you submit your (revised) paper to JMIR, please upload the PDF as supplementary file.

Don't worry if some text in the textboxes is cut off, as we still have the complete information in our database. Thank you!

Final step: Click submit !

Click submit so we have your answers in our database!

Submit Clear form

Never submit passwords through Google Forms.

This content is neither created nor endorsed by Google. Report Abuse - Terms of Service - Privacy Policy

Google Forms
